# Screening of the active Ingredients in Huanglian Jiedu decoction through amide bond-Immobilized magnetic nanoparticle-assisted cell membrane chromatography

**DOI:** 10.3389/fphar.2022.1087404

**Published:** 2022-12-19

**Authors:** Fengyun Liao, Dongmei He, Chi Teng Vong, Lisheng Wang, Zhangmei Chen, Tiejun Zhang, Hua Luo, Yitao Wang

**Affiliations:** ^1^ Macau Centre for Research and Development in Chinese Medicine, State Key Laboratory of Quality Research in Chinese Medicine, Institute of Chinese Medical Sciences, University of Macau, Macau, China; ^2^ The Fifth Affiliated Hospital, Southern Medical University, Guangzhou, Guangdong, China; ^3^ College of Chinese Material Medical, Guangzhou University of Chinese Medicine, Guangzhou, Guangdong, China; ^4^ Tianjin Engineering Laboratory of Quality Control Technology of Traditional Chinese Medicine, Tianjin Institute of Pharmaceutical Research, Tianjin, China

**Keywords:** Huanglian Jiedu decoction, Alzheimer’s disease, amide bond, magnetic nanoparticle-immobilized cell membranes, glutamate-induced toxicity, oxidative stress

## Abstract

**Introduction:** The Huanglian Jiedu decoction (HLJDD) is a Chinese herbal formula that exerts neuroprotective effects by alleviating oxidative stress injuries and may potentially be prescribed for treating Alzheimer’s disease; however, its active ingredients have not yet been identified. Cell membrane chromatography is a high-throughput method for screening active ingredients, but traditional cell membrane chromatography requires multiple centrifugation steps, which affects its separation efficiency. Magnetic nanoparticles are unparalleled in solid-liquid separation and can overcome the shortcomings of traditional cell membrane chromatography.

**Methods:** In this study, the neuroprotective effects of the components of HLJDD were screened through a novel magnetic nanoparticle-assisted cell membrane chromatography method. Magnetic nanoparticles and cell membranes were stably immobilized by amide bonds. Magnetic bead (MB)-immobilized cell membranes of HT-22 cells were incubated with the HLJDD extract to isolate specific binding components. The specific binding components were then identified by ultraperformance liquid chromatography (UPLC)—Orbitrap Fusion Tribrid MS after solid-phase extraction. The bioactivity of these components was analyzed in an HT-22 cellular model of glutamate-induced injury.

**Results and Discussion:** The preparation method of the composite of cell membrane and MBs has the advantages of simple preparation and no introduction of toxic organic reagents. MBs not only provide support for cell membranes, but also greatly improve the separation efficiency compared with traditional cell membrane chromatography. Fifteen of these components were found to specifically bind to the cell membranes, and seven of them were confirmed to reduce varying degrees of glutamate-induced toxicity in HT-22 cells. In conclusion, our findings suggest that the amide bond-based immobilization of magnetic nanoparticles on cell membranes, along with solid-phase extraction and UPLC, is an effective method for isolating and discovering the bioactive components of traditional Chinese medicines.

## Graphical Abstract

**Figure:**
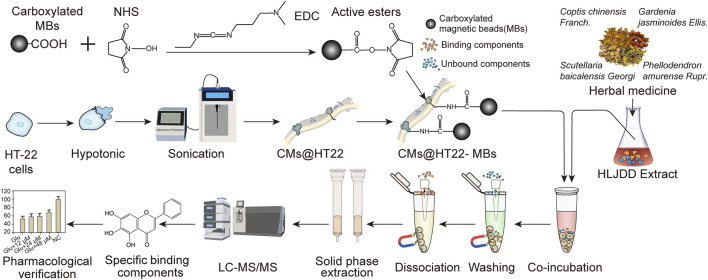

## 1 Introduction

Alzheimer’s disease (AD) is an irreversible neurodegenerative disease that causes memory impairment and cognitive decline. Currently, there are at least 50 million patients with AD worldwide. With the increasing aging population, the incidence of AD is increasing rapidly (Scheltens et al., 2016). Due to its high prevalence and disability rate, the long course of the disease, and high treatment costs, AD significantly impacts the patients’ families and society. However, effective treatments that can slow the progression of the disease are scarce (DeTure and Dickson, 2019; Scheltens et al., 2021).

The literature suggests that oxidative stress-related neuronal cell damage plays a vital role in the pathogenesis of AD (Cutler et al., 2004; Arimon et al., 2015; Tamagno et al., 2021). Oxidative stress leads to mitochondrial dysfunction and promotes the accumulation of amyloid beta in the brain, which induces neurotoxicity and triggers nerve cell death (Tofighi et al., 2021; Yang et al., 2021). Glutamate is a major excitatory neurotransmitter and an initiating factor in neuronal death in several neurodegenerative diseases (Ribeiro et al., 2017; Onaolapo and Onaolapo, 2021). Moreover, the high accumulation of reactive oxygen species is closely related to glutamate-induced neuronal damage (Pan et al., 2020; Tonsomboon et al., 2021). The Huanglian Jiedu decoction (HLJDD), which originates from “*Arcane Essentials from the Imperial Library*”, is composed of *Coptis chinensis* Franch (Ranunculaceae; Coptidis Rhizoma), *Scutellaria baicalensis* Georgi (Lamiaceae; Scutellariae Radix), *Phellodendron amurense* Rupr (Rutaceae; Phellodendri Amurensis cortex), and *Gardenia jasminoides* Ellis (Rubiaceae; Gardeniae fructus) in a ratio of 3:2:2:3 (He et al., 2022a; He et al., 2022b; Liao et al., 2022). As a Chinese classical formula with heat-clearing and detoxicating effects, HLJDD has been proved to have the potential to treat AD by many modern pharmacological studies (Sun et al., 2018; Wu et al., 2020). Numbers of clinical trials results also demonstrated that HLJDD has the effect of treating AD, including reducing the levels of β-amyloid, phosphorylated tau protein and inflammatory factors (Chen and Guan, 2016; Jeon et al., 2019), reducing the aggressiveness of dementia patients (Okamoto et al., 2013) and improving deviation and rejection behaviors (Qi et al., 2019; Urabe et al., 2020). Further pharmacological studies have shown that it reduces oxidative stress damage and exerts neuroprotective effects (Xu et al., 2000; Gu et al., 2021). However, the specific mechanisms underlying these effects are unknown.

In recent years, cell membrane (CM) chromatography has attracted widespread attention for elucidating the bioactive ingredients in TCMs; this method is based on the interactions between CM receptors and analytes. Although this technique provides high-throughput screening of potential bioactive ingredients in TCMs, it can be further improved. Traditional CM chromatography requires multi-step centrifugation to achieve effective solid–liquid separation during the washing of non-specific binding components and dissociated binding components. This process is time-consuming and might cause CM loss during processing. To overcome these limitations and improve the solid–liquid separation efficiency, magnetic nanoparticles have been introduced, which are unparalleled in solid–liquid separation (Hu et al., 2019a; Hu et al., 2019b; Sherwood et al., 2019). However, simpler preparation methods and other immobilization strategies need to be considered. Moreover, the ability of the composites of the magnetic nanomaterials and CMs to separate active components from more complex samples must be verified.

We hypothesized that it would be feasible to directly immobilize CMs with carboxylated magnetic nanoparticles through amide bonds, which could simplify the preparation process compared to those from previous studies. In addition, the fabricated composites can potentially be used to screen the active ingredients in TCM prescriptions composed of multiple medicinal materials.

This study developed a stable composite comprising CMs and magnetic beads (MBs) to screen the active ingredients in Chinese herbal compounds. First, HT-22 CMs were obtained, and a stable amide bond was formed between the amine group of the phospholipids and carboxylated MBs using *N*-ethyl-*N*′-(3-(dimethylamino) propyl) carbodiimide (EDC) and *N*-hydroxysuccinimide (NHS). The stability of the amide bond was studied using laser scanning confocal microscopy (LSCM), scanning electron microscopy (SEM), Fourier transform infrared spectroscopy (FTIR), transmission electron microscopy (TEM), vibrating sample magnetometry (VSM), and X-ray photoelectron spectroscopy (XPS). This composite material of MBs and CMs has the advantages of simple preparation process and no introduction of toxic organic reagent. Then, the stable composite material was successfully applied to screen the active ingredients in HLJDD. Due to the introduction of MBs, the separation efficiency of this method is greatly improved compared with traditional cell membrane chromatography. The establishment of this screening platform broadens the application of magnetic nanomaterials and demonstrates the feasibility of screening other TCM active ingredients.

## 2 Materials and methods

### 2.1 Materials

#### 2.1.1 Plant materials

The decoction pieces of HLJDD, namely, *C. chinensis* Franch (Ranunculaceae; Coptidis Rhizoma), *S. baicalensis* Georgi (Lamiaceae; Scutellariae Radix), *P. amurense* Rupr (Rutaceae; Phellodendri Amurensis cortex), and *G. jasminoides* Ellis (Rubiaceae; Gardeniae fructus), were purchased from Caizhilin Pharmaceutical Co., Ltd. (Guangzhou, China), and their origins were Sichuan, Hebei, Liaoning, and Jiangxi provinces, respectively. All the botanical drugs were verified by a qualified institution **(**Guangdong Hanchao Traditional Chinese Medicine Technology Co., Ltd. Guangzhou, China) according to the 2020 edition of the *Chinese Pharmacopoeia*.

#### 2.1.2 Chemicals materials

Standard substances, including oroxylin A-7-O-β-D-glucuronide, baicalin, dehydrocorydaline, wogonoside, worenine, 2,3,4,9-tetrahydro-1H-β-carboline-3-carboxylic acid, magnoflorine, phellodendrine, and palmatine, were provided by Chengdu Keloma Biotechnology Co., Ltd (Chengdu, China). Apigenin 7-O-glucuronide, dihydrochelerythrine, and groenlandicine were obtained from Chengdu Munster Biotechnology Co. Ltd. (Chengdu, China). Skullcapflavon II was purchased from Wuhan Tianzhi Biotechnology Co., Ltd. (Wuhan, China), and thalifendine was provided by Chengdu Pusi Biotechnology Co., Ltd. (Chengdu, China). Genistein 4′-O-glucuronide was purchased from Toronto Research Chemicals, Inc. (Toronto, Canada). The purity of all standard compounds was >98%. Oasis HLB SPE cartridges were provided by the Waters Corporation (Milford, MA, United States). Carboxylated MBs and Vitamin E (VE) were obtained from Shanghai Macklin Biochemical Co. Ltd (Shanghai, China). A bicinchoninic acid protein assay (BCA) kit was provided by Shanghai Enzyme Link Biotechnology Co., Ltd. (Shanghai, China). L-glutamic acid monosodium salt hydrate was purchased from Merck KGaA (Darmstadt, Germany). Dulbecco’s Modified Eagle’s Medium (DMEM) and fetal bovine serum (FBS) were obtained from GIBCO (Invitrogen Corporation, Carlsbad, CA, United States). Cell counting Kit-8 (CCK-8) was purchased from Dojindo Laboratories (Kumamoto, Japan). Acetonitrile (ACN) and formic acid (LC-MS grade) were procured by Thermo Fisher Scientific (New Jersey, United States).

### 2.2 Preparation of HLJDD

Rhizome of Coptidis Rhizoma (Ranunculaceae; *C. chinensis* Franch.), roots of Scutellariae Radix (Lamiaceae; *S. baicalensis* Georgi), bark of Phellodendri Amurensis cortex (Rutaceae; *P. amurense* Rupr.), and fruits of Gardeniae fructus (Rubiaceae, *G. jasminoides* Ellis.) decoction pieces with a total mass of 180 g in a ratio of 3:2:2:3 were weighed and soaked in water equivalent to 10 times (1,800 ml) the weight of the botanical drugs for 0.5 h and then refluxed for 1 h. Subsequently, the samples were filtered, and the residue was mixed in water equivalent to 8 times (1,080 ml) the weight of the botanical drugs and refluxed again for 1 h. The filtrates obtained after the first and second reflux processes were combined and concentrated to 250 ml *via* rotary evaporation. The extract is then freeze-dried into powder (Labconco, Kansas City, MO, United States). The qualitative and quantitative analysis of the extract of HLJDD was carried out. Qualitative analysis including ultraperformance liquid chromatography (UPLC) fingerprint and thin-layer chromatography (TLC) analysis method was established, and the UPLC fingerprint method implemented with different gradients, different stationary phases, and different detection wavelengths were verified. In addition, quantitative analysis was also carried out, and the three main components (geniposide, berberine, and baicalin) in HLJDD were determined as quality makers according to of the *Japanese Pharmacopoeia* (17 edition).

### 2.3 Cell culture and preparation of CMs@HT22

HT-22 cells were provided by Merck KGaA (Darmstadt, Germany) and cultured in a complete DMEM containing 10% FBS. The cells were then cultured in a cell incubator at 37°C. The CMs were prepared according to previously reported methods (He et al., 2022a; He et al., 2022b). The amount of CMs was examined using the BCA protein assay.

### 2.4 Preparation of the CMs@HT22-MB composite material

CMs@HT22-MB composites were prepared according to a previous report with minor modifications (Hu et al., 2019b). Subsequently, 400 mM NHS and 20 mM EDC were added to the carboxylated MB suspension (5 mg/mL). After that, the magnetic nanoparticles with active ester structures on their surface were separated using a magnetic field. The precipitates were washed with ultra-pure water three times, and residual reagents were removed. The precipitates were then vacuum-dried at 60°C for 12 h. To bind the magnetic nanoparticles with active ester structures to the CM surface, PBS and CMs (contains 5 mg total cell membrane protein) containing dispersed magnetic nanoparticles were mixed by ultrasonic waves at 4°C for 10 min. The mixture was then separated using an external magnetic field, and the excess CMs were discarded.

### 2.5 Characterization of CMs@HT22-MBs and MBs

CMs@HT22-MBs and MBs were incubated with fluorescein isothiocyanate-conjugated 1,2-distearoyl-*sn*-glycero-3-phosphoethanolamine polyethylene glycol (DSPE-PEG-FITC, molecular weight: 2,000) with the concentration of 1 mg/ml at 37°C for 30 min respectively, and then PBS were washed three times to remove excess dyes. The fluorescently labeled substances were then examined using LSCM (Leica TCS SP8, Leica, Allendale, NJ, United States). Moreover, the morphology of CMs@HT22-MBs and MBs was observed through TEM (FEI Tecnai G2 F20, Hillsboro, OR, United States) and SEM (Hitachi S-4800, Tokyo, Japan). Magnetic measurements were performed using VSM (SQUID-VSM; Quantum Design United Kingdom and Ireland, Leatherhead, United Kingdom). XPS (ESCALAB 250XI, Thermo Fisher Scientific, United States) was used to analyze the surface elemental composition of the obtained materials. FTIR spectra of the obtained materials were acquired on a Nicolet iS50 FTIR spectrometer (Thermo Fisher Scientific, United States) from 4,000 to 400 cm^−1^ to observe the changes in their functional groups.

### 2.6 Biospecific extraction of bioactive compounds from HLJDD

The lyophilized powder of CMs@HT22-MBs (10 mg) was resuspended in 2 mL PBS. Then, the HLJDD extract (5 mL, 0.78 mg/mL) was added and incubated at 37°C for 1 h. The control group was composed of CMs@HT22-MBs treated with PBS. Then, under an external magnetic field, the composite material combined with the potential active ingredient was rinsed eight times with 5 mL PBS to remove the non-specifically bound components. Subsequently, 5 mL citrate-sodium citrate buffer (pH 4.0) was added, and the resultant solution was incubated for 30 min to dissociate the specific binding components from the CM. The dissociated solution was collected and fractionated by SPE, according to a previously reported method (He et al., 2022a; He et al., 2022b). This sample was used for mass spectrometry (MS) analysis.

### 2.7 Identification of bound components with LC-MS

The chromatographic separation was conducted on a Vanquish™ Flex Ultra Performance LC (UPLC) system (Thermo Fisher Scientific, Milford, MA, United States) equipped with an Orbitrap Fusion Tribrid TM high-definition mass spectrometer (Thermo Fisher Scientific) and a Hypersil GOLD C18 column (2.1 × 100 mm, 1.9 μm). The separation was carried out at a flow rate of 0.3 mL/min at 35°C using a mobile phase system composed of ACN (A) and 0.2% formic acid–water (B). The gradient elution steps were as follows: 0–1 min, 5% A; 1–5 min, 5%–26% A; 5–9 min, 26%–28% A; 9–12 min, 28%–98% A; 12–15 min, 98% A. The injection volume was 3 μl.

Electrospray ionization was employed and operated in both negative and positive ion modes. A full-scan mode from m/z 100 to 1,000 was used. The core parameters of the mass spectrometer were as follows: the spraying voltage was 2,000 V and 3,000 V, respectively, in positive and negative ion modes; the ion transfer tube temperature, 350°C; vaporizer temperature, 300°C; auxiliary gas (N_2_), 10 respective arbitrary units; sweep gas (N_2_), one respective arbitrary unit; sheath gas (N_2_), 40 respective arbitrary units; The Compound Discoverer 3.1.0.305 software (Thermo Fisher Scientific, Milford, MA, United States) was used to analyze the MS data with a retention time of 0.2 min and a quality tolerance of 5 ppm.

### 2.8 Cell viability assay

The CCK-8 kit was used to evaluate the protective effect of compounds against glutamate-induced cell damage in HT-22 cells. The cells were seeded into 96-well plates at 3 × 10^3^ cells/well density and incubated at 37°C for 24 h. They were then treated with different concentrations of phellodendrine, magnoflorine, 2,3,4,9-tetrahydro-1H-β-carboline-3-carboxylic acid, groenlandicine, berberrubine, apigenin 7-O-glucuronide, worenine, palmatine, genistein 4′-O-glucuronide, dehydrocorydaline, baicalin, wogonoside, oroxylin A-7-O-β-D-glucuronide, skullcapflavon II, and VE (400 μM, positive control) for 12 h before being exposed to 7 mM L-glutamate for 24 h. The medium was then replaced with 100 μl serum-free DMEM containing 10% CCK-8 reagent. The cells were then incubated at 37°C for 2 h. The absorbance of each well was measured at 450 nm with a microplate reader. The experiment was repeated three times and the data were expressed as the mean ± standard deviation. One-way ANOVA was conducted using SPSS (version 20.0; IBM, New York, NY, United States). The level of significance was set at *p* <0.05.

## 3 Results

### 3.1 Quality control of HLJDD samples

HLJDD was prepared using botanical drugs from different origins, and the batches of medicinal materials were analyzed by UPLC and TLC. The results showed that 15 batches of HLJDD samples exhibited similar chromatographic behavior, indicating that the components in each batch of the samples were relatively stable (Supplementary Figures S1–S6; Supplementary Table S1). The sample preparation and analysis showed good reproducibility.

### 3.2 Preparation and characterization of CMs@HT22-MBs composite material

**FIGURE 1:**
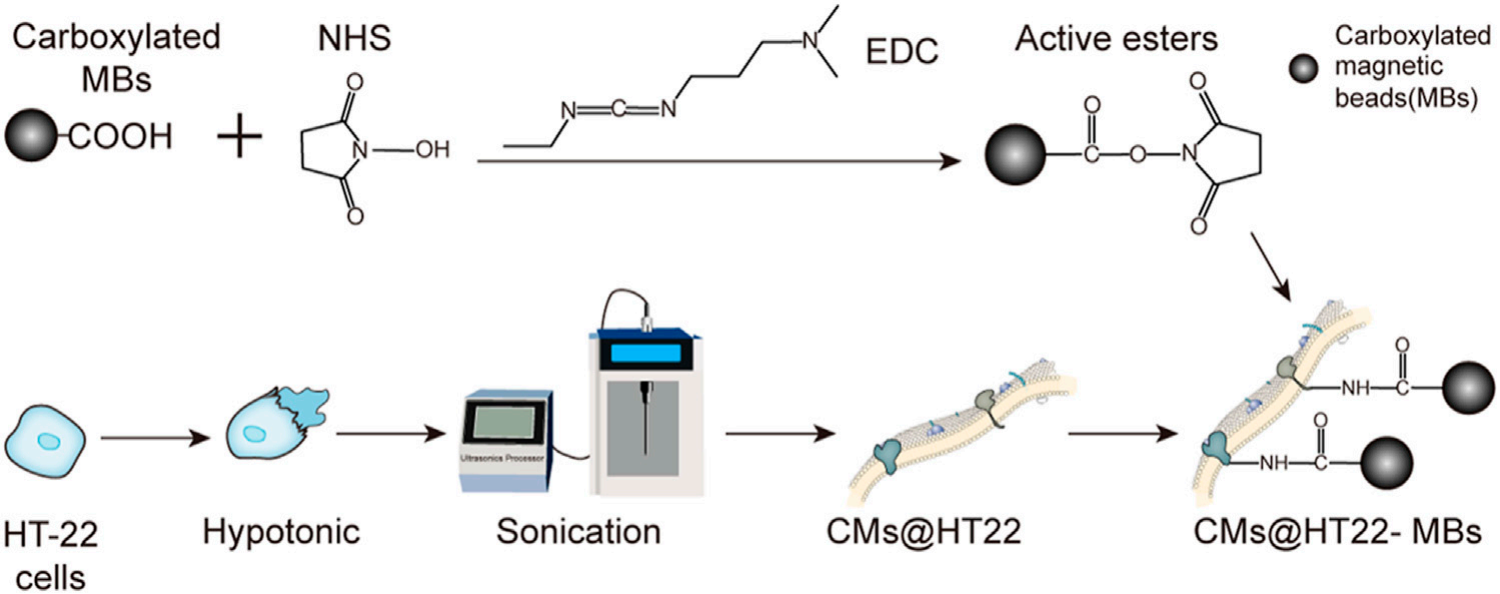
Preparation of membrane-coated magnetic beads (CMs@HT22-MBs). First, active esters were generated using the carboxylated magnetic nanoparticles through the reaction of EDC and NHS. Then, pure HT-22 cell membranes (CMs) were prepared by cell lysis and multiple centrifugations. Finally, the active ester immobilized with the magnetic nanoparticles reacted with the abundant amino groups on the cell membrane to form amide bonds, and a stable composite material was developed.

The preparation of stable magnetic nanoparticle-immobilized CM composites is shown in Figure 1. This material was then applied to screening the active ingredients in Chinese herbal medicines. We used EDC/NHS to modify the carboxylated MBs to generate active ester-rich intermediates. The HT22 CM fragments were simultaneously prepared by ultrasonication, which comprised phospholipid bilayers and provided abundant free amino groups. Afterwards, MBs containing the active ester groups were reacted with the amino group of the CM, establishing a stable CMs@HT22-MBs composite material through amide bond formation.

**FIGURE 2:**
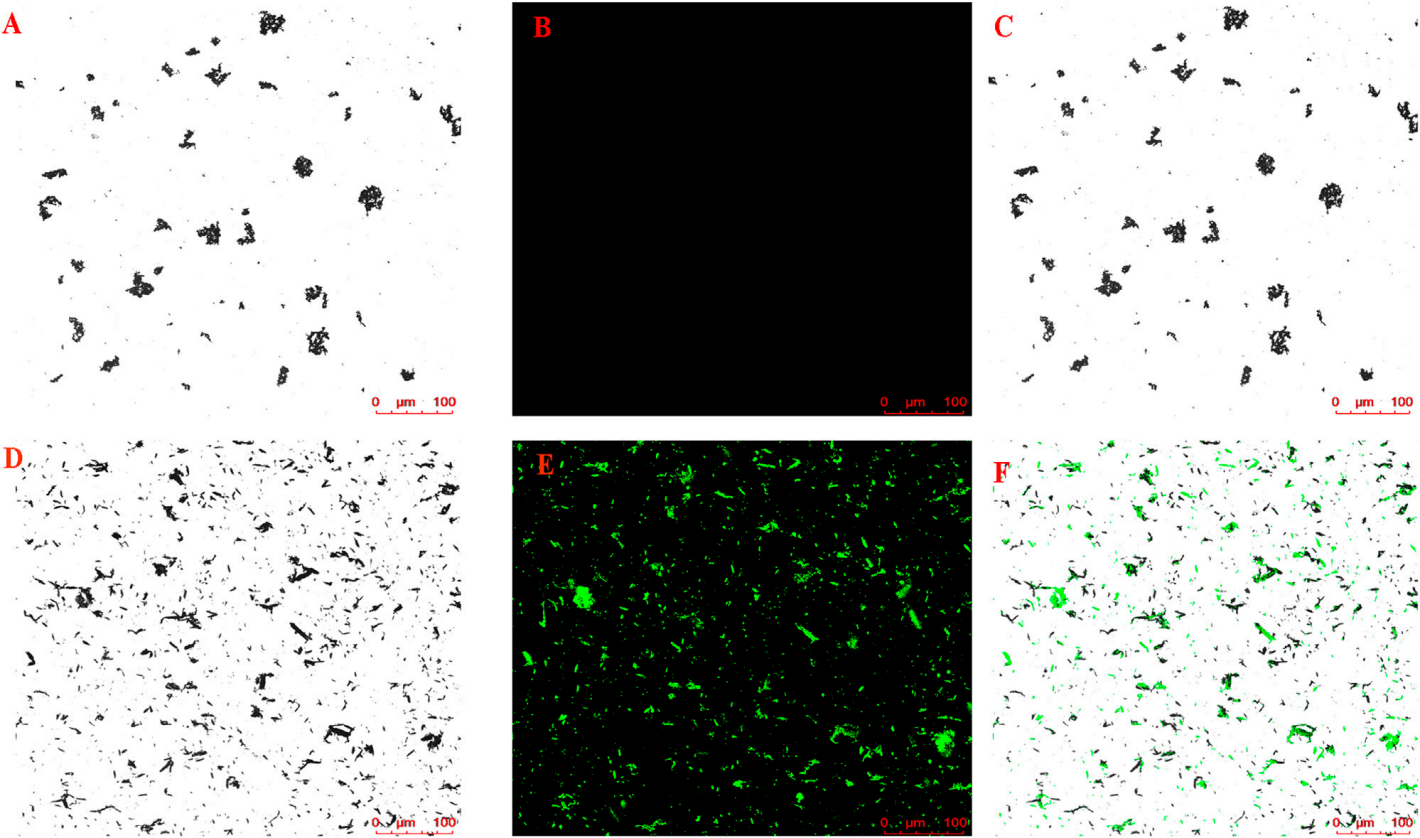
Confocal microscopic images of **(A–C)** CMs@HT22-MBs and **(D–F)** MBs. CMs@HT22-MBs and MBs were incubated with fluorescein isothiocyanate-conjugated 1,2-distearoyl-*sn*-glycero-3-phosphoethanolamine polyethylene glycol (DSPE-PEG-FITC, molecular weight: 2,000) with a concentration of 1 mg/ml at 37°C for 30 min respectively, and then PBS were washed three times to remove excess dyes. The fluorescently labeled substances were then examined using laser scanning confocal microscopy laser scanning confocal microscopy. **(A,D)** Brightfield. **(B,E)** Excitation at 488 nm to visualize DSPE-PEG-FITC-labeled CMs. **(C)** Merged images of A and B and **(F)** D and E.

The magnetic nanoparticle-immobilized CM was incubated with the HLJDD extract to screen the potential neuroprotective ingredients that could specifically bind to the HT-22 CM receptors. After labeling the CMs@HT22-MBs composites and MBs with FITC, LSCM was used to visually verify whether the magnetic nanoparticles were successfully bound to the surface of the CM. The labeled CMs@HT22-MBs composite material showed obvious green fluorescence (Figures 2D–F). However, no green-fluorescent signal was detected on the MB surface (Figures 2A–C), indicating that the magnetic nanoparticles were successfully bound to the surface of the CM.

**FIGURE 3:**
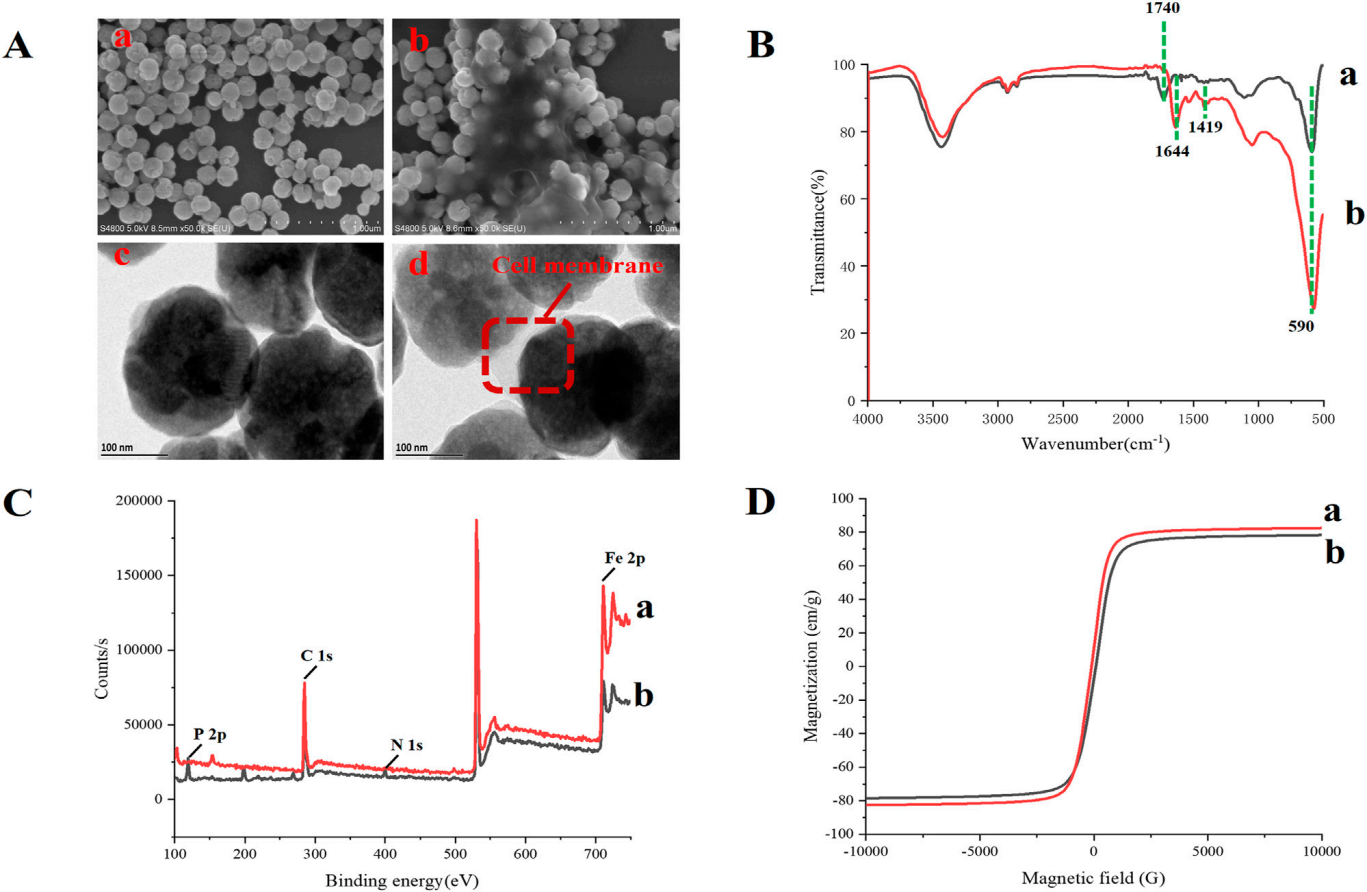
Characterization of CMs@HT22-MBs and MBs. The morphology of CMs@HT22-MBs and MBs was observed through transmission electron microscopy (TEM) and scanning electron microscopy (SEM). Fourier transform infrared spectroscopy (FTIR) spectra of the obtained materials were acquired on a FTIR spectrometer from 4,000 to 400 cm^−1^ to observe the changes in their functional groups. X-ray photoelectron spectroscopy (XPS) was used to analyze the surface elemental composition of the obtained materials. Magnetic measurements were performed using vibrating sample magnetometry (VSM). **(A)** SEM images of MB (a) and CMs@HT22-MBs (b) ; TEM images of MB (a) and CMs@HT22-MBs (b). **(B)** FTIR spectra of (a) MBs and (b) CMs@HT22-MBs. **(C)** XPS of (a) MBs and **(B)** CMs@HT22-MBs. **(D)** Magnetic hysteresis loops of (a) MBs and (b) CMs@HT22-MBs.

TEM and SEM were used to verify whether MBs were successfully immobilized with the CM. This was done by determining the differences in the morphological characteristics of MBs and CMs@HT22-MBs. The results showed that the CMs were successfully immobilized with the magnetic nanoparticles (Figure 3A). The FTIR spectra of the MBs (curve a) and CMs@HT22-MBs (curve b) are shown in Figure 3B.

peak at 1,740 cm^−1^ (curve a) is ascribed to the tensile vibrations of the C=O bond. Curve b shows the immobilized spectra of the CMs, including the disappearance of the spectral band at 1,740 cm^−1^ and the appearance of two spectral bands at 1,644 cm^−1^ and 1,419 cm^−1^, respectively, caused by the stretching vibration of C–O and C–N bonds in CMs@HT22-MBs. This observation confirmed the formation of an amide bond between the CMs and carboxylated MBs. Moreover, the reduction in the intensity of the band at 580 cm^−1^ (the tensile vibration of the tetrahedron site of Fe_3_O_4_) further demonstrated the effective fixation of the CMs with functional magnetic nanoparticles. XPS was used to study the elements and surface groups of the carboxylated MBs and CMs@HT22-MBs. As shown in Figure 3C, Fe 2p and C 1s peaks were observed in the XPS spectra of the carboxylated MBs (curve a), confirming that the commercialized magnetic nanoparticles have a -COOH structure on the surface. N 1s and P 2P peaks, which corresponded to the nitrogen and phosphorus atoms on the CM, respectively, appeared on curve b after the CMs were coated with magnetic nanoparticles. This suggests that the preparation of CMs@HT22-MBs was successful. Furthermore, the magnetic properties of the CMs@HT22-MBs (Figures 3D b) and MBs (Figures 3D a) were investigated through VSM. Due to the presence of CMs, the saturation magnetization value (78.6 emu g^−1^) in the CMs@HT22-MBs was lower than that in MBs (83.9 emu g^−1^). However, the CMs@HT22-MBs still retain enough magnetic activity to support the solid–liquid separation of complex systems.

### 3.3 Biospecific extraction and identification of the bioactive components in HLJDD

**FIGURE 4:**
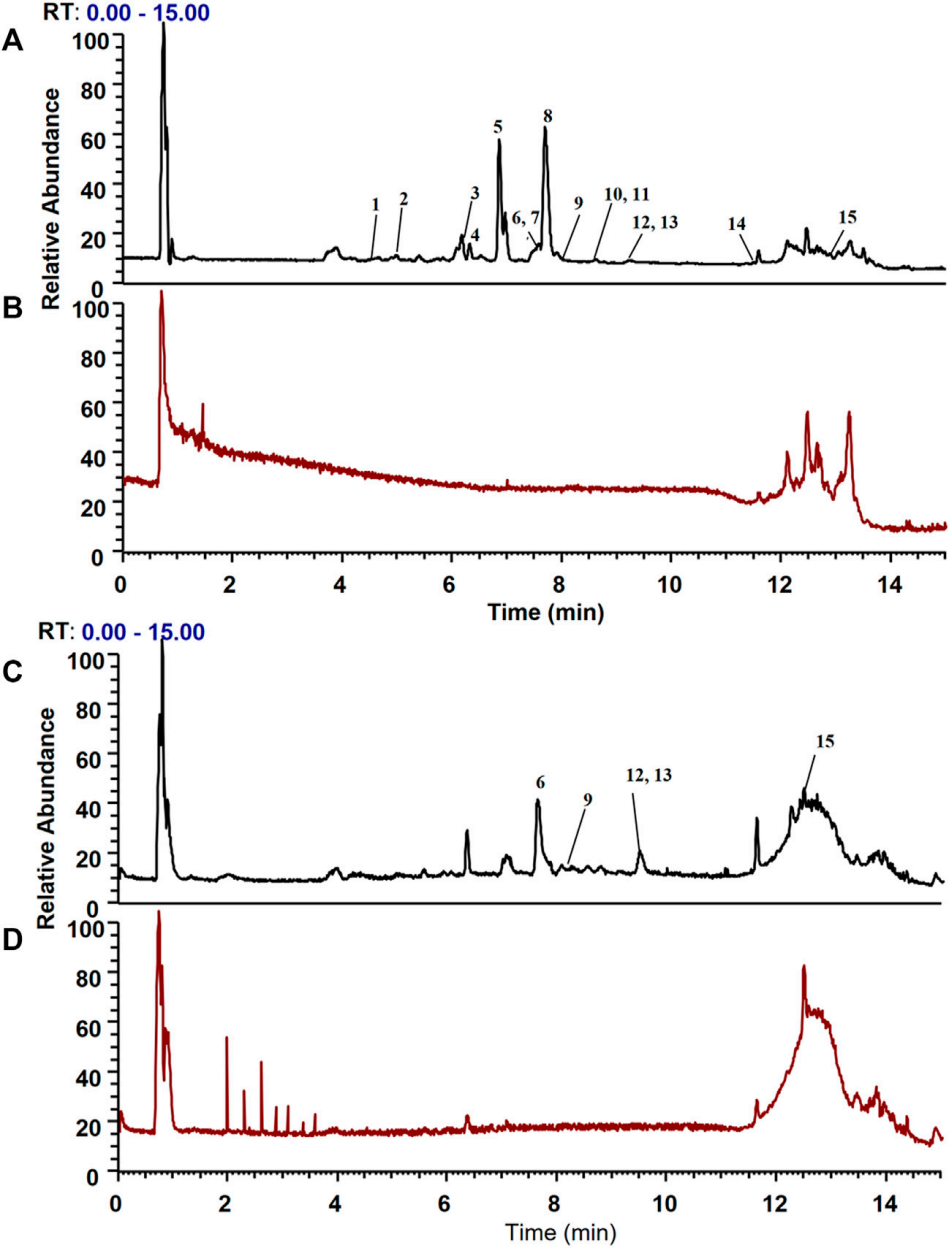
Total ion chromatograms of the dissociated solutions of the Huanglian Jiedu decoction (HLJDD) treatment and control groups in positive and negative modes. The dissociated components in the solution were identified using the UPLC-Orbitrap Fusion Tribrid MS system, and 15 different components were identified in the solution of the HLJDD-treated group compared with the control group in positive and negative ion modes. **(A)** Positive ion mode of the HLJDD group; **(B)** positive ion mode of the control group; **(C)** negative ion mode of the HLJDD group; **(D)** negative ion mode of the control group.

**FIGURE 5:**
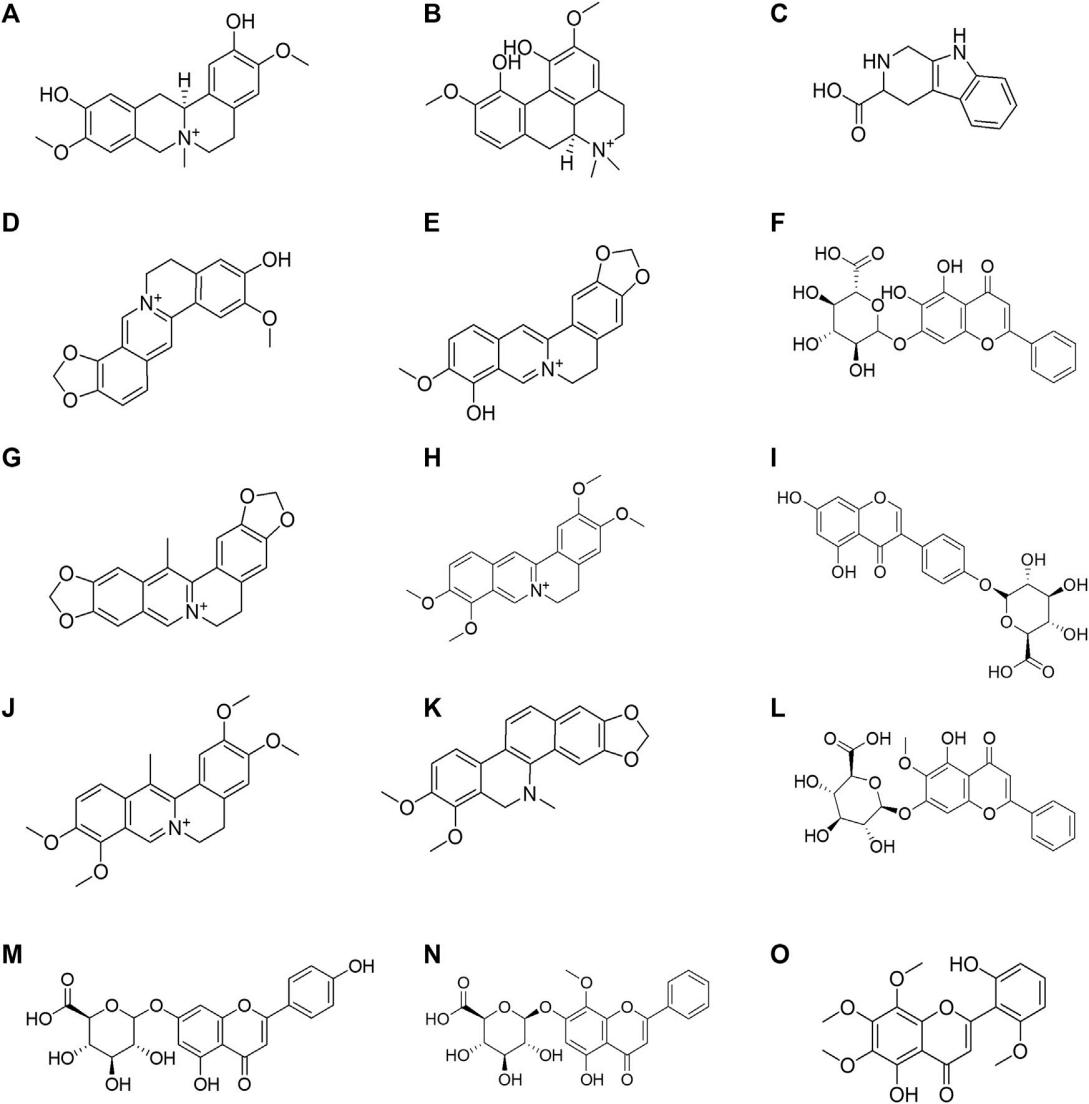
Structural formula of the 15 Huanglian Jiedu decoction (HLJDD) components dissociated from the CMs@HT22-MBs. **(A)** phellodendrine; **(B)** magnoflorine; **(C)** 2,3,4,9-tetrahydro-1H-β-carboline-3-carboxylic acid; **(D)** groenlandicine; **(E)** berberrubine; **(F)** baicalin; **(G)** worenine; **(H)** palmatine; **(I)** genistein 4′-O-glucuronide; **(J)** dehydrocorydaline; **(K)** dihydrochelerythrine; **(L)** oroxylin A-7-O-β-D-glucuronide; **(M)** apigenin 7-O-glucuronide; **(N)** wogonoside; **(O)** skullcapflavon II.

CMs@HT22-MBs were co-incubated with HLJDD extracts to isolate the potential bioactive components. Subsequently, the specific binding components are dissociated from the CM and collected. The dissociated components in the solution were identified using the UPLC-Orbitrap Fusion Tribrid MS system, and 15 different components were identified in the solution of the HLJDD-treated group compared with the control group in positive and negative ion modes (Figure 4). Incubation of HLJDD (or PBS) with active ester containing MB was set as a negative control to verify whether the ingredients in HLJDD were non-specifically bound to active ester not fixed by amide bond on cell membrane. Results shown that no negative interference on the specific binding of CMs@HT22-MBs to HLJDD (Supplementary Figure S7). As shown in Figure 5, Supplementary Figures S8, S9, and Table 1, 15 compounds, including phellodendrine, magnoflorine, 2,3,4,9-Tetrahydro-1H-β-carboline-3-carboxylic acid, groenlandicine, berberrubine, apigenin 7-O-glucuronide, worenine, palmatine, genistein 4′-O-glucuronide, dehydrocorydaline, baicalin, oroxylin A-7-O-β-D-glucuronide, wogonoside, and skullcapflavon II, were preliminarily identified based on the retention times, fragmentation patterns, previous publications, and standard products. Therefore, these compounds were preliminarily identified as potential active ingredients that could specifically bind to the HT-22 CM receptors.

**TABLE 1:** Identification of the specific binding component of Huanglian Jiedu decoction by UPLC-Orbitrap Fusion TMS.

NO.	RT min	Ion Mode	Measured m/z	Predicted m/z	Formula	Name	MS^2^	Mass error (ppm)
1	4.52	[M]^+^	342.16937	342.17108	C_20_H_24_NO_4_	phellodendrine	342.16959, 192.10181	5.00
2	4.97	[M]^+^	342.1694	342.17108	C_20_H_24_NO_4_	(+)-magnoflorine	342.16986, 297.11209, 265.08597	4.91
3	6.11	[M + H]^+^	217.1069	217.09715	C_12_H_12_N_2_O_2_	2,3,4,9-tetrahydro-1H-β-carboline-3-carboxylic acid	217.10690, 173.08060, 155.07002, 111.04387, 101.05955, 83.04907, 55.05424	−4.49
4	6.17	[M]^+^	322.10678	322.10738	C_19_H_16_NO_4_^+^	groenlandicine	322.10715, 307.08371, 279.08887	1.86
5	7.26	[M]^+^	322.10696	322.10738	C_19_H_16_NO_4_^+^	berberrubine	322.10727, 307.08386, 279.08881	1.30
6	7.48	[M + H]^+^	447.09137	447.09219	C_21_H_18_O_11_	baicalin	447.09213, 271.06006	1.83
	7.58	[M-H]^-^	445.07724	445.07763	C_21_H_18_O_11_	baicalin	269.04544, 175.02425, 113.02376, 85.02889	0.89
7	7.50	[M]^+^	334.10681	334.10848	C_20_H_16_NO_4_^+^	worenine	344.1073, 306.11221	5.00
8	7.66	[M]^+^	352.15375	352.15543	C_21_H_22_NO_4_+	palmatine	352.15421, 336.12271, 308.12787	4.77
9	8.08	[M + H]^+^	447.09134	447.09219	C_21_H_18_O_11_	genistein 4′-O-glucuronide	447.09198, 271.06009	1.90
	8.24	[M-H]^-^	445.07745	445.07763	C_21_H_18_O_11_	genistein 4′-O-glucuronide	269.04547, 175.02431, 113.02377, 85.02891	0.41
10	8.43	[M]^+^	366.16946	366.16998	C_22_H_24_NO_4_^+^	dehydrocorydaline	366.16980, 350.13867, 322.14362	1.43
11	8.61	[M + H]^+^	350.13806	350.13868	C_21_H_19_NO_4_	dihydrochelerythrine	350.13846, 335.11490, 306.11218	1.78
12	8.74	[M-H]-	459.09317	459.09328	C_22_H_20_O_11_	oroxylin A-7-O-β-D-glucuronide	268.03738, 175.02415, 113.02375, 85.02891	0.25
13	9.13	[M + H]^+^	447.09152	447.09219	C_21_H_18_O_11_	apigenin 7-O-glucuronide	447.09210, 285.07565, 271.06009	1.49
	9.35	[M-H]^-^	445.07758	445.07763	C_21_H_18_O_11_	apigenin 7-O-glucuronide	269.04517, 175.02423, 113.02374, 85.02894	0.12
14	9.27	[M + H]^+^	461.10696	461.10784	C_22_H_20_O_11_	wogonoside	461.10791, 285.07556,270.05209	1.90
	9.48	[M-H]^-^	459.09326	459.09328	C_22_H_20_O_11_	wogonoside	283.06137, 268.03781,175.02437,113.02381,85.02895	0.05
15	12.20	[M + H]^+^	375.10687	375.10744	C_19_H_18_O_8_	skullcapflavon II	375.10699, 345.06000, 327.04932, 265.17984, 227.05473, 100.07552	1.53
	12.22	[M-H]^-^	373.09271	373.09289	C_19_H_18_O_8_	skullcapflavon II	373.09241, 343.04541, 328.02173, 194.99,289, 169.01346	0.48

RT, retention time; MS2, second stage of mass spectrometry; TMS, tribrid mass spectrometry.

### 3.4 Verification of the pharmacological activities of enriched compounds using glutamate-induced HT-22 cells

**FIGURE 6:**
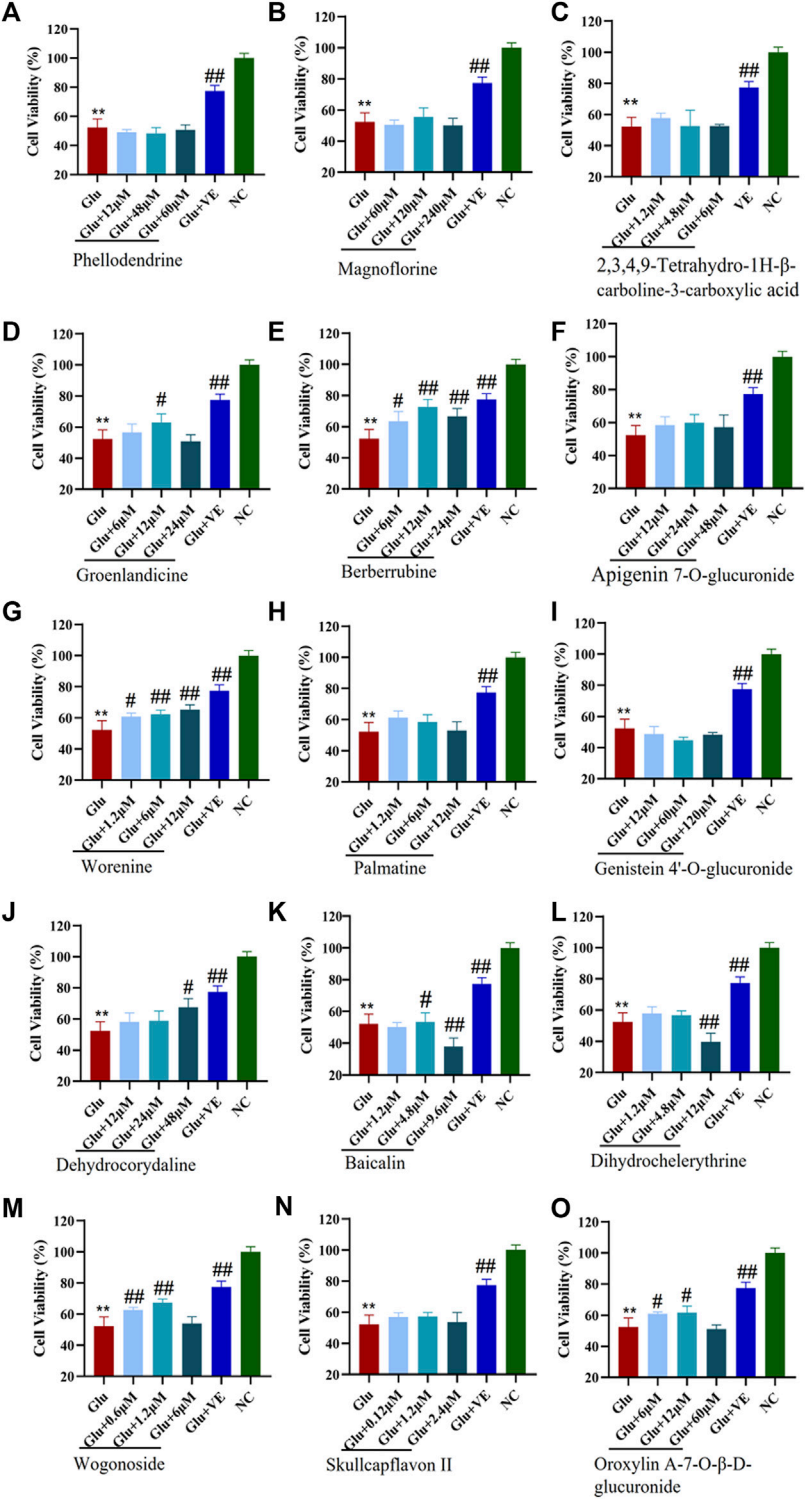
Protective effects of CMs@HT22-MB-specific binding components on glutamate-induced toxic damage in HT-22 cells. The CCK-8 kit was used to evaluate the protective effect of compounds against glutamate-induced cell damage in HT-22 cells. The cells were treated with different concentrations of specific binding components or VE (positive control) for 12 h before being exposed to 7 mML-glutamate for 24 h. The medium was then replaced with 100 μl serum-free DMEM containing 10% CCK-8 reagent. The cells were then incubated at 37°C for 2 h. The absorbance of each well was measured at 450 nm with a microplate reader. **(A)** phellodendrine; **(B)** magnoflorine; **(C)** 2,3,4,9-tetrahydro-1H-β-carboline-3-carboxylic acid; **(D)** groenlandicine; **(E)** berberrubine; **(F)** apigenin 7-O-glucuronide; **(G)** worenine; **(H)** palmatine; **(I)** genistein 4’-O-glucuronide; **(J)** dehydrocorydaline; **(K)** baicalin; **(L)** dihydrochelerythrine; **(M)** wogonoside; **(N)** skullcapflavon II; **(O)** oroxylin A-7-O-β-D-glucuronide. ^*^ *p* < 0.05 and ^**^ *p* < 0.01 *vs.* normal control group (NC); ^#^ *p* < 0.05 and ^##^ *p* < 0.01 *vs.* glutamate-treated group (Glu).

The CCK-8 viability assay was used to examine the protective effects of specific binding components on glutamate-induced HT-22 cells. We first investigated the toxicity of glutamate by evaluating its viability in the HT-22 cells. In this experiment, the median lethal dose (LD_50_) of glutamate for HT-22 cells was 7.0 mM (Supplementary Figure S10). Therefore, we used 7 mM of glutamate in the subsequent construction of an oxidative stress cell model of HT-22 cells. As shown in Figure 6, the survival rates of the HT-22 cells decreased significantly (*p* <0.01) after being treated with 7 mM glutamate. This suggests that the glutamate-induced neurotoxicity model was successfully established. Different concentrations of groenlandicine, berberrubine, worenine, dehydrocorydaline, oroxylin A-7-O-β-D-glucuronide significantly increased the viability of glutamate-treated HT-22 cells (*p* <0.05, *p* <0.01). Cell viability was significantly improved after VE treatment (*p* <0.01). Phellodendrine, magnoflorine, 2,3,4,9-tetrahydro-1H-β-carboline-3-carboxylic acid, apigenin 7-O-glucuronide, palmatine, genistein 4′-O-glucuronide, dihydrochelerythrine, skullcapflavon II and wogonoside did not exhibit any protective effect against glutamate in HT-22 cells. For baicalin, the survival rate of cells increased significantly after the intervention with 4.8 μM concentration. However, after a high concentration of baicalin (9.6 μM), the survival rate of the cells was significantly decreased compared with that of the model group, suggesting that high concentration of baicalin had a certain degree of cytotoxicity to the model cells. Similarly, after the intervention of high concentration of dihydrochelerythrine (12 μM), the cell survival rate was significantly decreased compared with the model group, suggesting that the overdose of dihydrochelerythrine may cause some toxicity in glutamate-treated HT-22 cells.

## 4 Discussion

We successfully developed a composite system comprising magnetic nanoparticles and CMs using amide bonds. This method was used for screening the active components of HLJDD that exert neuroprotective effects. Fifteen components that were specifically bound to the HT-22 CM were successfully isolated from HLJDD and identified. Seven of these components exhibited different degrees of activity in reducing glutamate-induced toxicity. Thus, the combination of CMs@HT22-MBs, SPE, and LC-MS was successfully established to screen and identify the bioactive components in HLJDD. This approach can serve as a screening platform for potential active ingredients in other TCMs.

Identifying the active ingredients in TCM is essential for quality control and drug development. CM chromatography has attracted increasing attention due to its high screening efficiency and specificity in elucidating the active ingredients of TCMs (Liao et al., 2018a; Liao et al., 2018b; He et al., 2022a; He et al., 2022b). However, this method has several limitations. For instance, the low solid–liquid separation efficiency of this method makes it extremely time-consuming. Researchers have been looking for a suitable alternative method to overcome these limitations.

Notably, MBs have many remarkable advantages, such as easy preparation, good compatibility, and efficient solid–liquid separation. Currently, there are two common strategies for immobilizing MBs and CMs. One of them is based on physical methods (Sherwood et al., 2019; Xu et al., 2019; Qi et al., 2020; He et al., 2022a; He et al., 2022b). Although this method is simple and the receptor activity on the CM is well preserved during its course, the separation of the CMs from the MBs is performed under a strong external magnetic field, which may result in instability. The other method involves the stable binding of CMs and MBs through chemical bonds (Hu et al., 2019a; Hu et al., 2019b). This greatly improves the stability of the CM and MB composite system. However, the preparation methods for these composite systems reported in the literature are highly complicated. Therefore, simpler preparation methods and other immobilization strategies need to be considered. Moreover, the ability of the composites of the magnetic nanomaterials and cell membranes to separate active components from more complex samples must be verified.

In this study, a simple method for stably combining cell membranes and magnetic nanoparticles based on amide bond was successfully constructed, and the constructed composite system was characterized by means of different angles. The results confirmed that the cell membrane and magnetic nanoparticles were effectively fixed. Then the complex system has been further tried to obtain the components of the complex system of TCMs. In this study, eight washes with PBS solution were performed to remove non-specifically bound components. As for traditional CM chromatography, this step usually requires multiple centrifugations, and each centrifugation takes about 15 min, making it a time-consuming process. Under the action of an external magnetic field, the CM with the immobilized magnetic nanoparticles was completely separated from the washing solution in only 10 s each time. Therefore, the constructed magnetic nanoparticle-assisted screening system shortens the separation time to at least 60 times less than that of the traditional CM chromatography method. The results showed that this method has obvious advantages compared with the traditional cell membrane chromatography method. The introduction of magnetic beads effectively shortens the washing time and dissociation steps, and improves the analysis efficiency. In view of the stable combination of chemical bonds, the composite system can still remain stable under the action of an external magnetic field, providing support for the cell membrane. In addition, because no more toxic organic reagents are used in the synthesis process, the activity of cell membrane receptors is largely preserved.

Therefore, we newly established a magnetic nanoparticle-assisted screening system based on amide bond immobilization. Compared with the previous literature reports, this method has the advantage of easier and more reliable preparation. Due to the introduction of magnetic nanoparticles, the separation efficiency of this method is greatly improved compared with traditional cell membrane chromatography. Using the composite of magnetic nanoparticles and HT22 cell membranes, 15 components that bind to the cell membrane of HT-22 were successfully screened. Therefore, the magnetic nanoparticle-cell membrane composite system constructed in this paper can be used for efficient screening of effective components in TCMs, and it is expected to be used for component separation in other complex systems.

To date, there is no effective treatment for AD, which is largely due to its complex pathogenesis. The early “Aβ hypothesis” and “tau hypothesis” were widely recognized at one time, but many drugs targeting Aβ and tau did not achieve the expected clinical efficacy, indicating that those two hypotheses cannot fully explain the pathogenesis of AD. Abundant evidence demonstrated that oxidative stress is involved in the pathological process of AD, which could lead to neuronal apoptosis and accelerate the generation of AD (Kam et al., 2019; Chen et al., 2020). Previous studies have shown that HLJDD can play a role in treating AD by reducing oxidative stress damage (Xu et al., 2000; Gu et al., 2021). However, the specific mechanisms underlying these effects are unknown. Therefore, we chose to elucidate the active ingredients of HLJDD in the treatment of AD from the perspective of reducing damage caused by oxidative stress.

Glutamate, an important excitatory neurotransmitter in the brain, plays a key role in learning and memory (William and McEnteet, 1993). However, numerous studies have demonstrated that excess glutamate is highly involved in the development of AD (Coyle and Puttfarcken, 1993; Choi et al., 2018), including the induction of excitotoxicity and oxidative stress injury. When excess glutamate binds to ionic glutamate receptors (iGluRs), excitatory toxicity is generated and extracellular Ca^2+^ influx is triggered, thus inducing the process of apoptosis (Hynd et al., 2004; He et al., 2013). On the other hand, high levels of glutamate could cause cystine/glutamate antiporter dysfunction, resulting in decreased levels of intracellular cysteine and glutathione, triggering antioxidant dysfunction (Latif et al., 2022). Accompanied by the increase of reactive oxygen species and the generation of lipid peroxidation, oxidative stress damage is induced, leading to cell death of neuronal cells (Phung, et al., 2020). Because hippocampal neuronal HT-22 cells do not express iGluR, their glutamate-induced excitotoxicity is negligible (Sato et al., 2016; Phung, et al., 2020). Therefore, glutamate-treated HT-22 cells are widely used as ideal models for model for oxidative stress injury (Bonaterra et al., 2017; Sugano et al., 2020). In the present study, a glutamate-induced cell damage model was used to evaluate specific binding components. Among the specific binding components, groenlandicine, berberrubine, worenine, dehydrocorydaline, baicalin, oroxylin A-7-O-β-D-glucuronide, and wogonoside significantly reduced glutamate-induced cell damage. Groenlandicine (Jung et al., 2009), dehydrocorydaline (Li et al., 2021; Lin et al., 2022), baicalin (Chen et al., 2015; Ding et al., 2015; Zhao et al., 2018; Yu et al., 2022), and wogonoside (Yang et al., 2014) have been reported to have neuroprotective effects against excitotoxic cell death. Herein, the protective effects of berberrubine, worenine, and oroxylin A-7-O-β-D-glucuronide in oxidative stress injuries have been reported for the first time to the best of our knowledge.

Here, we developed a composite system comprising magnetic nanoparticles and cell membranes using amide bonds for screening the active components of HLJDD that exert neuroprotective effects. Although this newly established method has many advantages over traditional CM chromatography, such as improved analysis efficiency and stable fixation of CMs and magnetic nanoparticles, some components might activate intracellular pathways after penetrating cell membrane instead of binding with membrane receptors. In addition, the active metabolites of compounds might be another way to protect cells (Yang et al., 2017), not the prototype of chemicals. Moreover, conducting pharmaco-dynamic evaluation and mechanistic studies of the active ingredients discovered in this study is still essential. Undoubtedly, verifying the activity of these active ingredients on other AD cell models (such as Aβ and tau protein induced cell damage) is also worth exploring, which will also be our follow-up work.

## Data Availability

The original contributions presented in the study are included in the article/Supplementary Material, further inquiries can be directed to the corresponding authors.

## Abbreviations

AD, Alzheimer’s disease; CM, Cell Membrane; CCK-8, Cell counting Kit-8; EDC, N-ethyl-N′-(3-(dimethylamino) propyl) carbodiimide; FTIR, Fourier transform infrared spectroscopy; HLJDD, Huanglian Jiedu decoction; LSCM, Laser scanning confocal microscopy; NHS, N-hydroxysuccinimide; SEM, Scanning electron microscopy; TEM, Transmission electron microscopy (TEM); UPLC, Ultraperformance liquid chromatography; VSM, Vibrating sample magnetometry; XPS, X-ray photoelectron spectroscopy (XPS).
