# Supplementary material for: Screening of the active Ingredients in Huanglian Jiedu decoction through amide bond-Immobilized magnetic nanoparticle-assisted cell membrane chromatography
Source: Front Pharmacol. 2022 Dec 19;13:1087404. doi: 10.3389/fphar.2022.1087404 (PMC9837740; doi:10.3389/fphar.2022.1087404)
Supplement: Supplementary file 1 [file DataSheet1.pdf]

frontiers

Supplementary Material

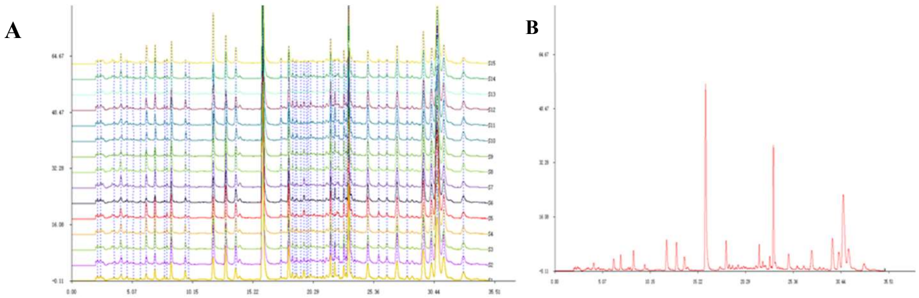

Supplementary Figure 1. Qualitative analysis of Huanglian Jiedu decoction (HLJDD). (A) fingerprints of 15 batches of samples of HLJDD; (B) reference fingerprint of HLJDD. The chromatographic separation was conducted on an Ultra High Performance Liquid Chromatography system equipped with a Photo Diode Array Detector and a Acquity UPLC HSS T3 column (2.1 × 100 mm, 1.8 µm). The separation was carried out at a flow rate of 0.3 mL/min at 40 °C using a mobile phase system composed of ACN (A) and 10mM ammonium acetate–water (acetic acid to adjust pH to 4.0) (B). The gradient elution steps were as follows: 0–15 min, 5-22% A; 15–28 min, 22–70% A; 28–30 min, 70–100% A. The injection volume was 5 µL and the detection wavelength was 250nm. The results shown that the quality of 15 batches of HLJDD samples were stable.

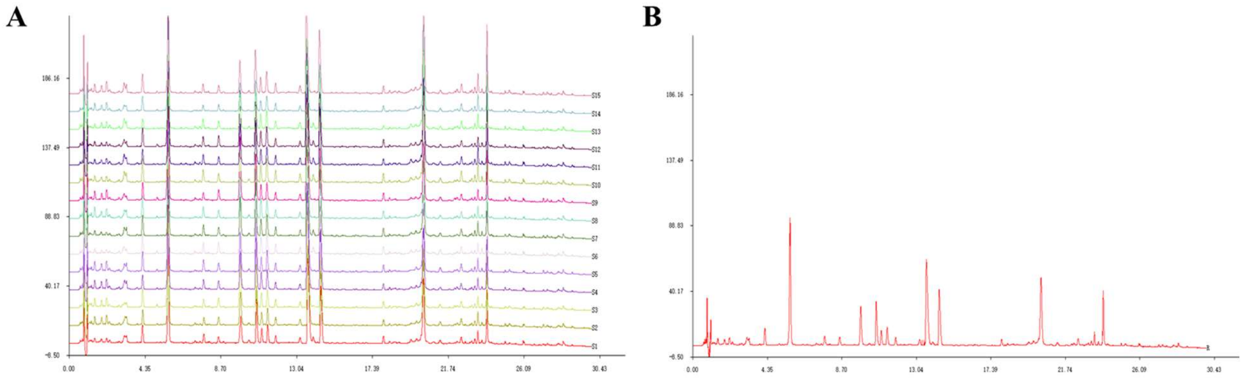

Supplementary Figure 2. Qualitative analysis of Huanglian Jiedu decoction (HLJDD). (A) fingerprints of 15 batches of samples of HLJDD; (B) reference fingerprint of HLJDD. The chromatographic separation was conducted on an Ultra High Performance Liquid Chromatography system equipped with a Photo Diode Array Detector and a Acquity UPLC CSH C18 column (2.1 × 100 mm, 1.7 μm). The separation was carried out at a flow rate of 0.3 mL/min at 40 °C using a mobile phase system composed of ACN (A) and 0.1% formic acid–water (B). The gradient elution steps were as follows: 0–20 min, 8-23% A; 20–30min, 23–70% A. The injection volume was 5 μL and the detection wavelength was 250nm. The results shown that the quality of 15 batches of HLJDD samples were stable.

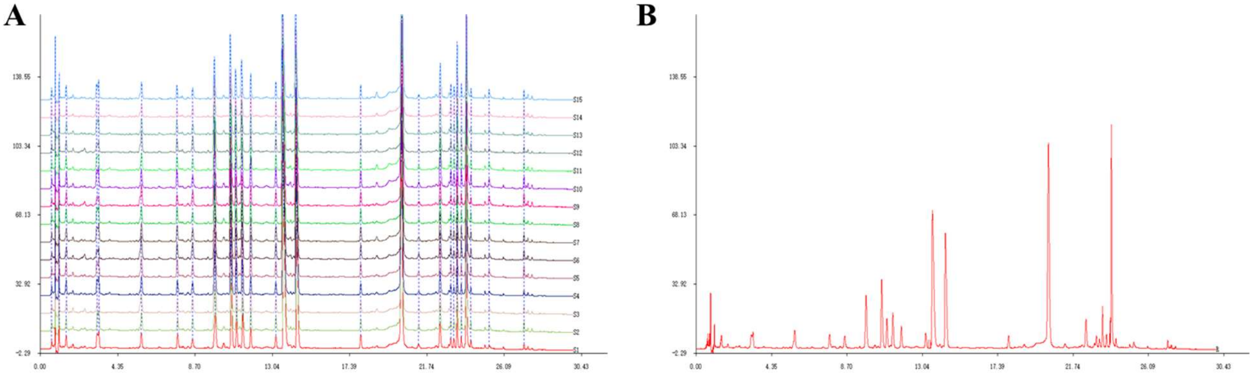

Supplementary Figure 3. Qualitative analysis of Huanglian Jiedu decoction (HLJDD). (A) fingerprints of 15 batches of samples of HLJDD; (B) reference fingerprint of HLJDD. The chromatographic separation was conducted on an Ultra High Performance Liquid Chromatography system equipped with a Photo Diode Array Detector and a Acquity UPLC CSH C18 column (2.1 × 100 mm, 1.7 μm). The separation was carried out at a flow rate of 0.3 mL/min at 40 °C using a mobile phase system composed of ACN (A) and 0.1% formic acid–water (B). The gradient elution steps were as follows: 0–20 min, 8-23% A; 20–30min, 23–70% A. The injection volume was 5 μL and the detection wavelength was 280nm. The results shown that the quality of 15 batches of HLJDD samples were stable.

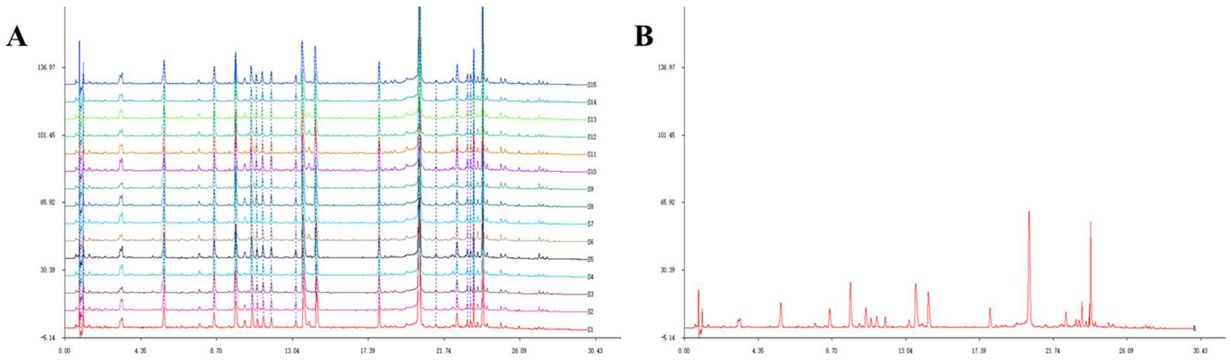

Supplementary Figure 4. Qualitative analysis of Huanglian Jiedu decoction (HLJDD). (A) fingerprints of 15 batches of samples of HLJDD; (B) reference fingerprint of HLJDD. The chromatographic separation was conducted on an Ultra High Performance Liquid Chromatography system equipped with a Photo Diode Array Detector and a Acquity UPLC CSH C18 column (2.1 × 100 mm, 1.7 μm). The separation was carried out at a flow rate of 0.3 mL/min at 40 °C using a mobile phase system composed of ACN (A) and 0.1% formic acid–water (B). The gradient elution steps were as follows: 0–20 min, 8-23% A; 20–30min, 23–70% A. The injection volume was 5 μL and the detection wavelength was 300nm. The results shown that the quality of 15 batches of HLJDD samples were stable.

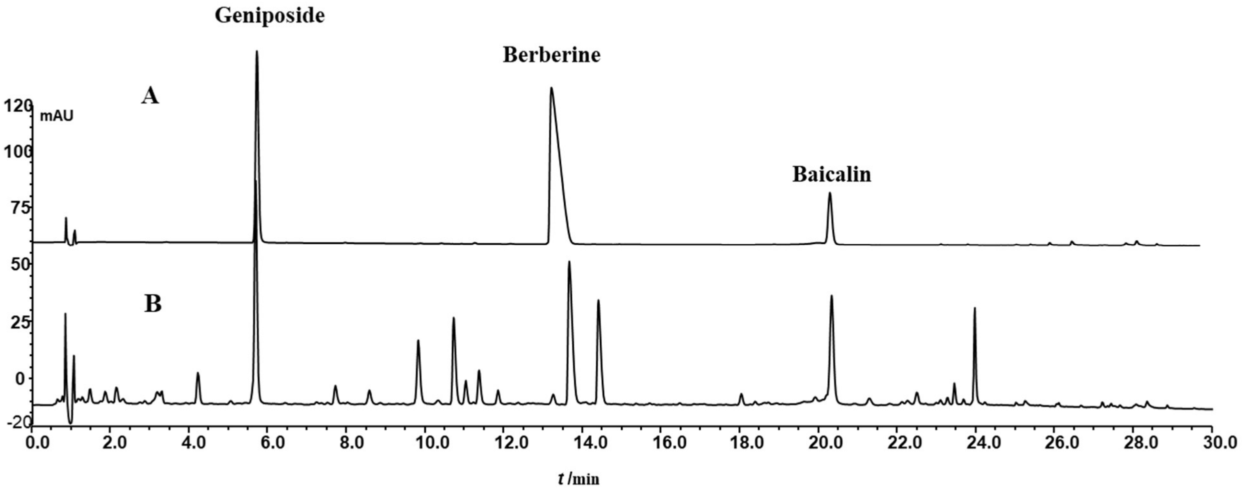

Supplementary Figure 5. Quantitative analysis of geniposide, berberine and baicalin in the extract of Huanglianjiedu decoction (HLJDD). (A) Mixed Standard; (B) HLJDD extract. The chromatographic separation was conducted on an Ultra High Performance Liquid Chromatography system equipped with a Photo Diode Array Detector and a Acquity UPLC CSH C18 column (2.1 × 100 mm, 1.7 μm). The separation was carried out at a flow rate of 0.3 mL/min at 40 °C using a mobile phase system composed of ACN (A) and 0.1% formic acid–water (B). The gradient elution steps were as follows: 0–20 min, 8-23% A; 20–30min, 23–70% A. The injection volume was 5 μL and the detection wavelength was 250nm.

Supplementary Table 1. Quantitative analysis of geniposide, berberine and baicalin in the extract of Huanglianjiedu decoction (HLJDD)

Batch	Geniposide(mg/g)	Berberine (mg/g)	Baicalin (mg/g)
1	37.30	13.18	72.24
2	31.98	12.16	74.06
3	41.50	15.56	64.06
4	31.64	16.35	56.90
5	39.81	14.17	75.79
6	43.76	18.75	52.70
7	38.71	14.18	72.96
8	41.08	13.63	61.11
9	37.90	17.45	54.22
10	46.95	11.64	95.14
11	41.53	14.05	82.58
12	41.96	18.24	51.45
13	36.60	14.29	73.30
14	31.02	9.52	84.96
15	36.59	14.19	91.37

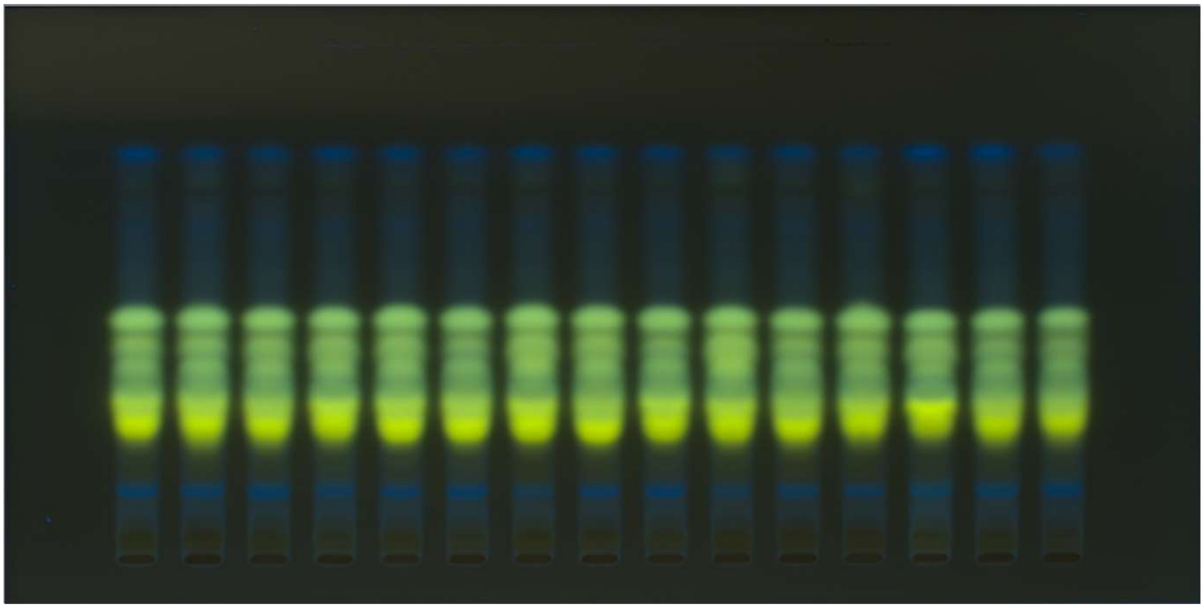

Supplementary Figure 6. Thin layer chromatography (TLC) of 15 batches of samples of Huanglian Jiedu Decoction (HLJDD). Shake 15 mg of freeze-dried powder of HLJDD with 1mL of 50% methanol-water, centrifuge, and use the supernatant liquid as the sample solution. According to the guidance of TLC, samples 15 batches of HLJDD were used for testing. Spot 10 µL each of the sample solution on a plate of silica gel for thin-layer chromatography. Develop the plate with a mixture of ethyl acetate, ammonia solution and methanol (5:1:1) to a distance of about 8 cm, and air-dry the plate. Examine under ultraviolet light (main wavelength: 365 nm). The results shown that the quality of 15 batches of HLJDD samples was stable.

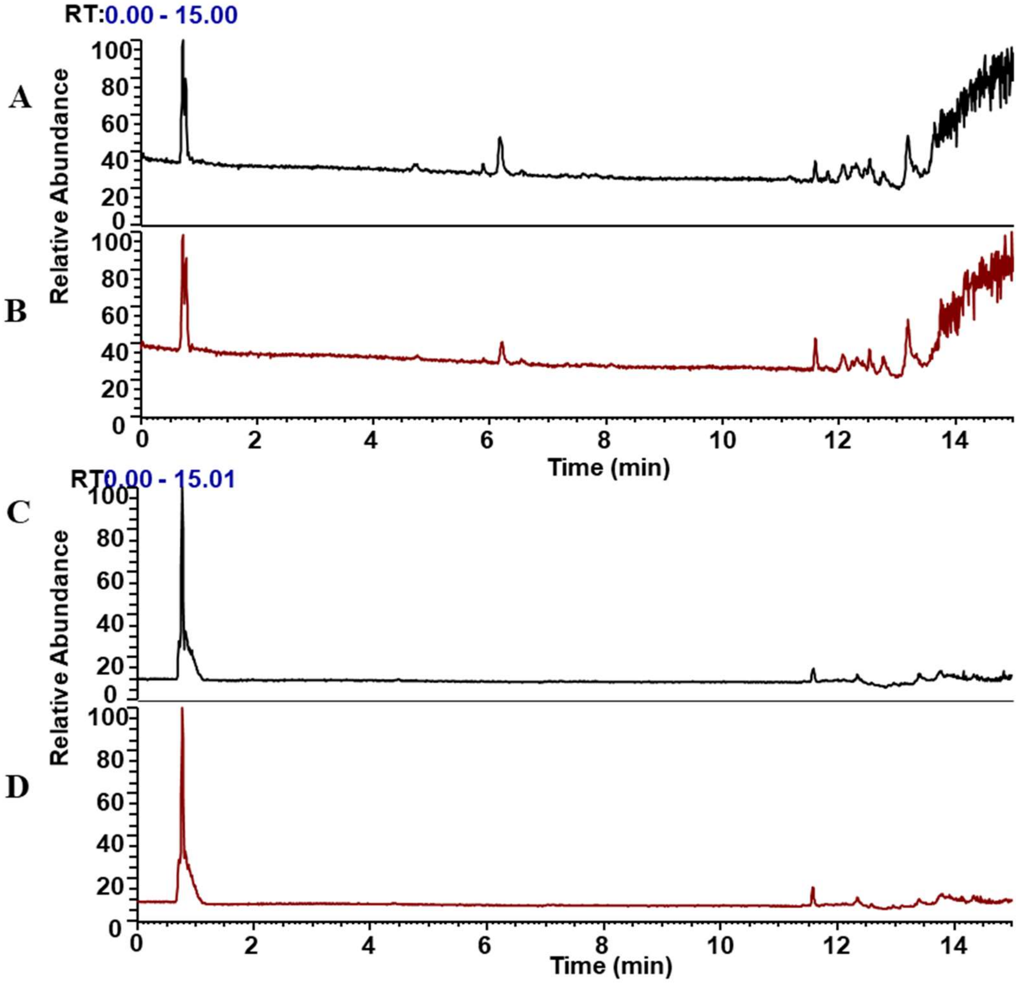

Supplementary Figure 7. Total ion chromatograms of the dissociated solutions after incubation of active ester with Huanglian Jiedu Decoction (HLJDD) or PBS in positive and negative modes. Incubation of HLJDD with active ester containing MB was set as a negative control to verify whether the ingredients in HLJDD were non-specifically bound to active ester not fixed by amide bond on cell membrane. The dissociated components in the solution were identified using the UPLC-Orbitrap Fusion Tribrid MS system. There was no negative interference on the specific binding of CMs@HT22-MBs to HLJDD. **(A)** Positive ion mode of the HLJDD group; **(B)** positive ion mode of the control group; **(C)** negative ion mode of the HLJDD group; **(D)** negative ion mode of the control group.

Supplementary Figure 8. Mass spectra of the 15 Huanglian Jiedu decoction (HLJDD) components dissociated from the CMs@HT22-MBs

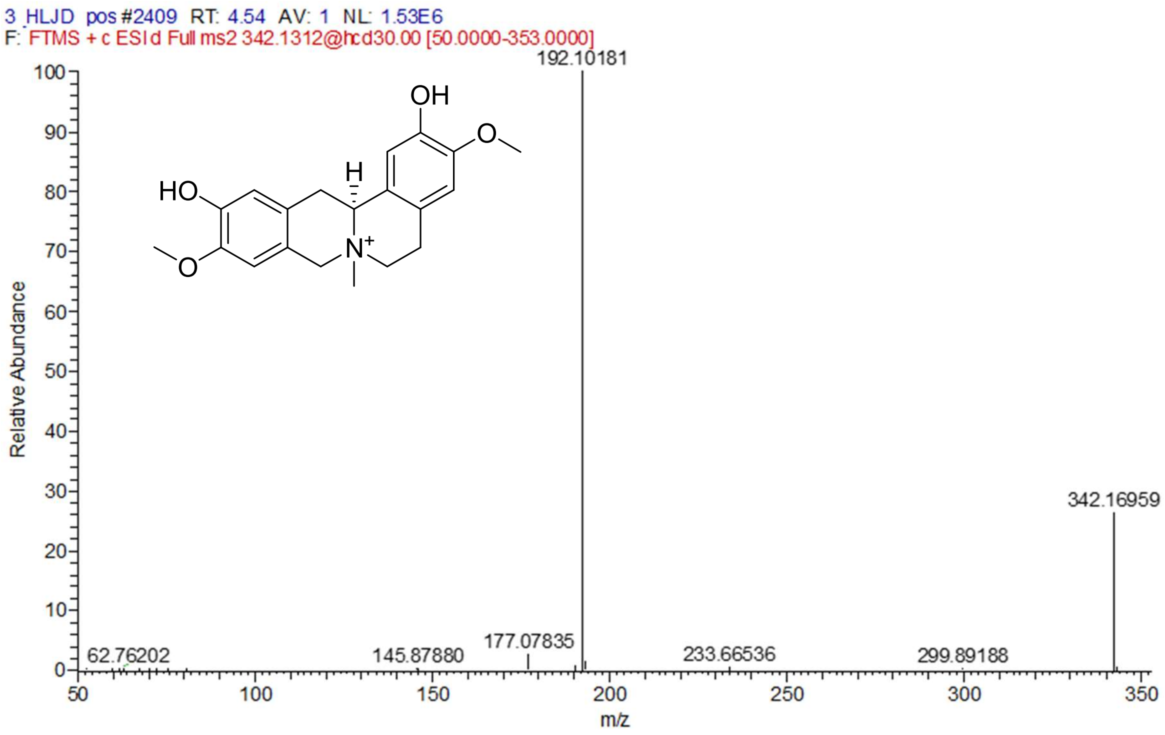

(A) Positive mode spectra for phellodendrine

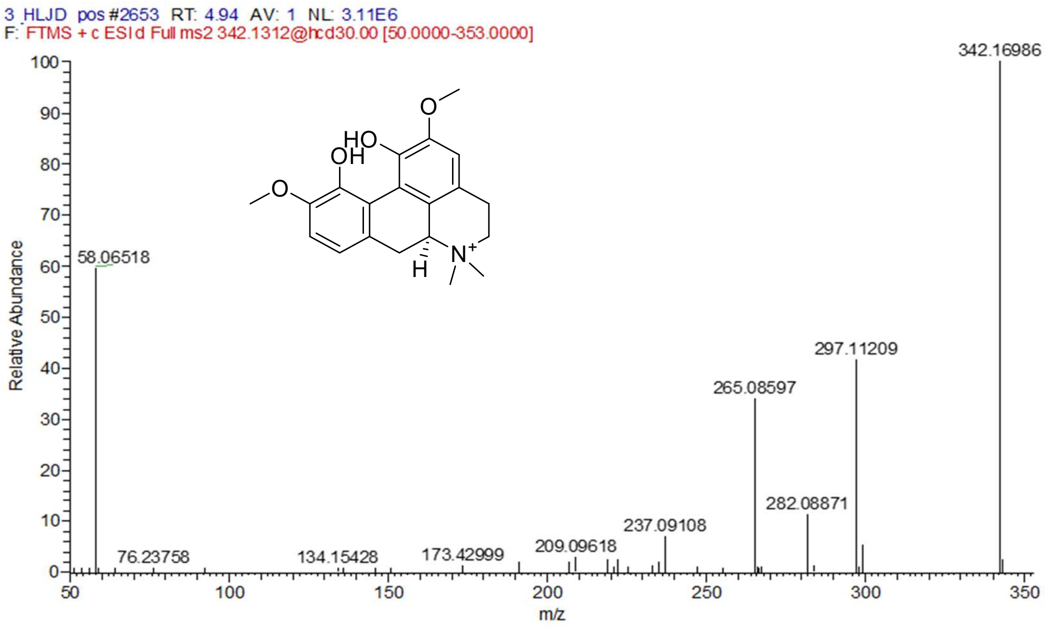

(B) Positive mode spectra for magnoflorine

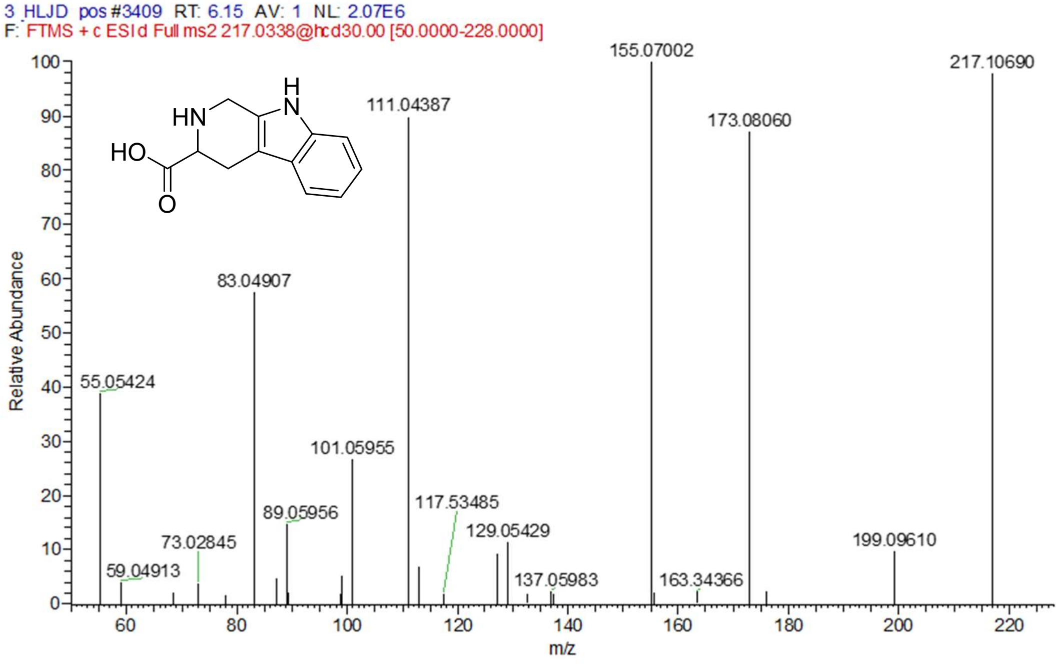

(C) Positive mode spectra for 2,3,4,9-tetrahydro-1H-β-carboline-3-carboxylic acid

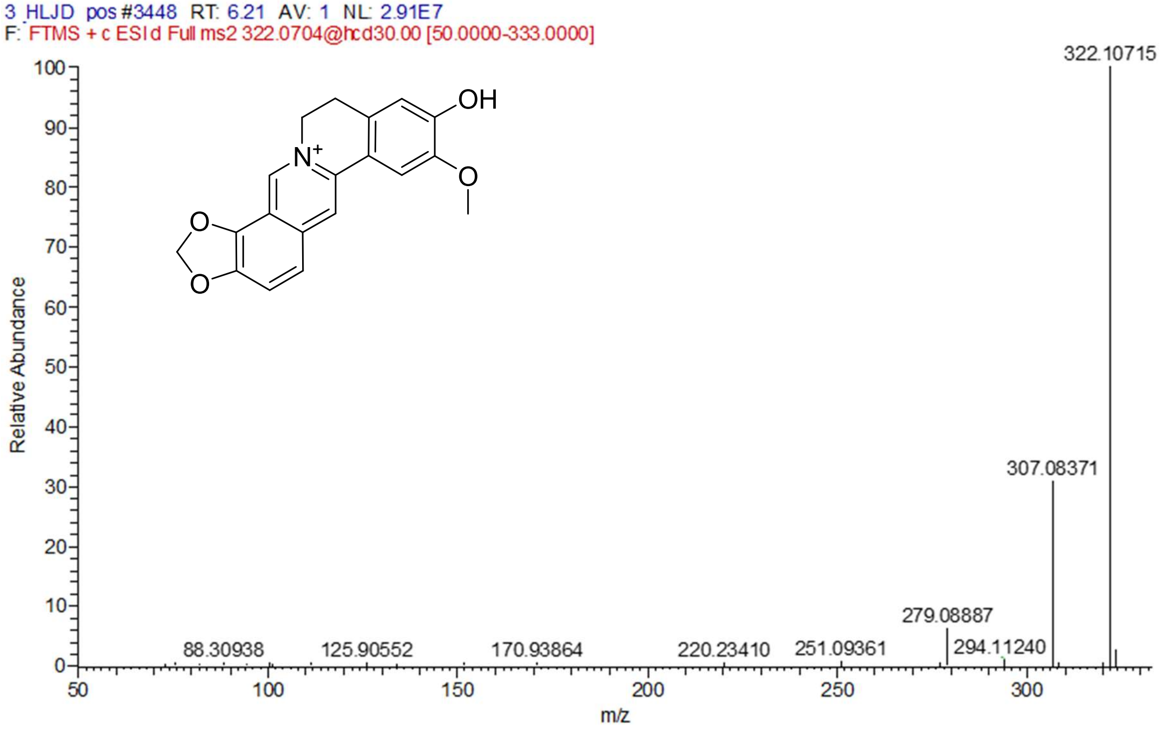

(D) Positive mode spectra for groenlandicine

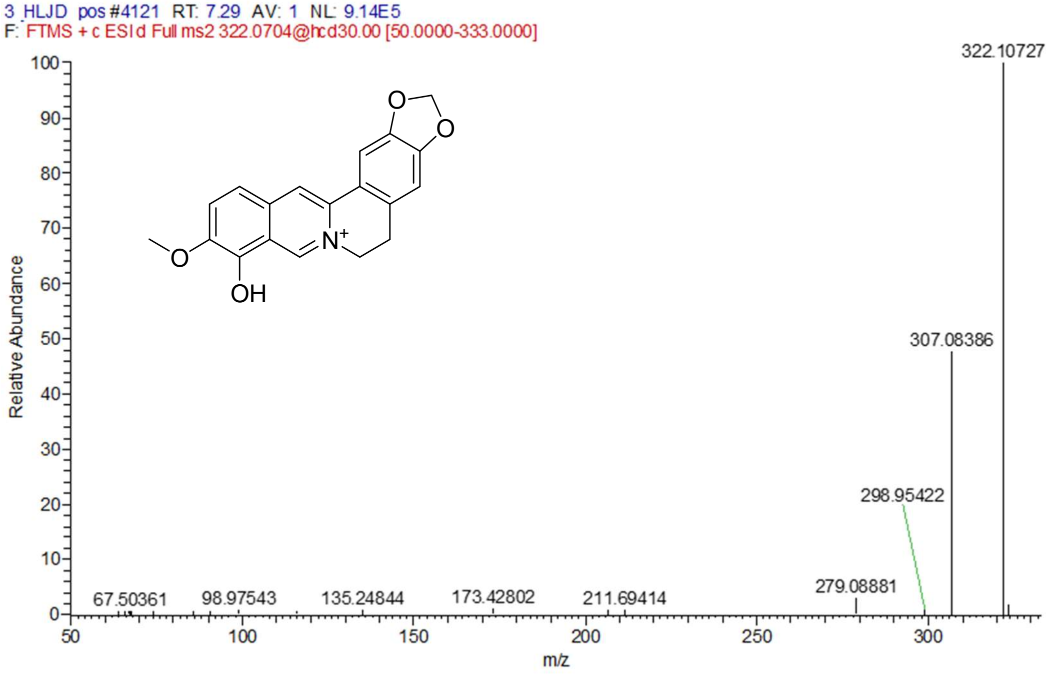

(E) Positive mode spectra for berberrubine

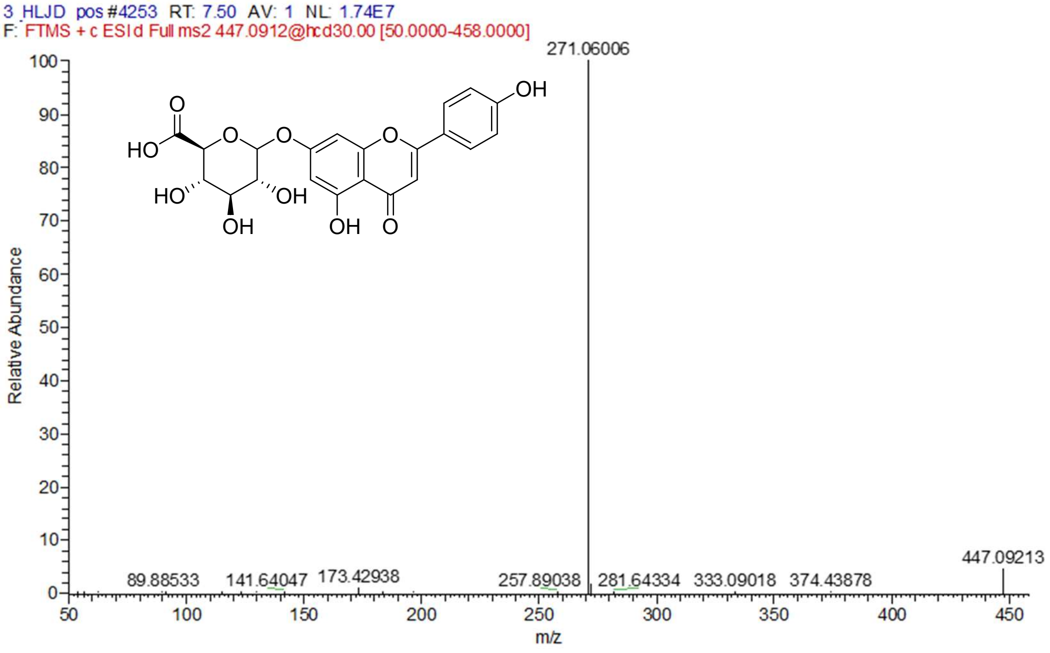

(F) Positive mode spectra for baicalin

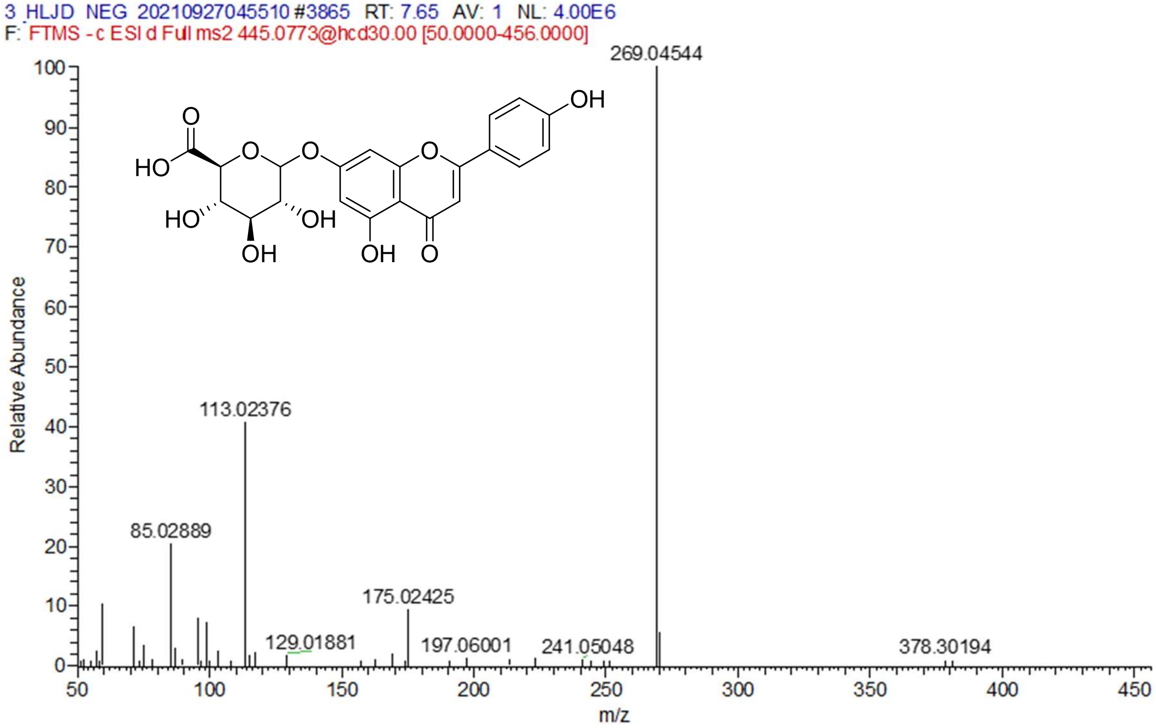

(G) Negative mode spectra for baicalin

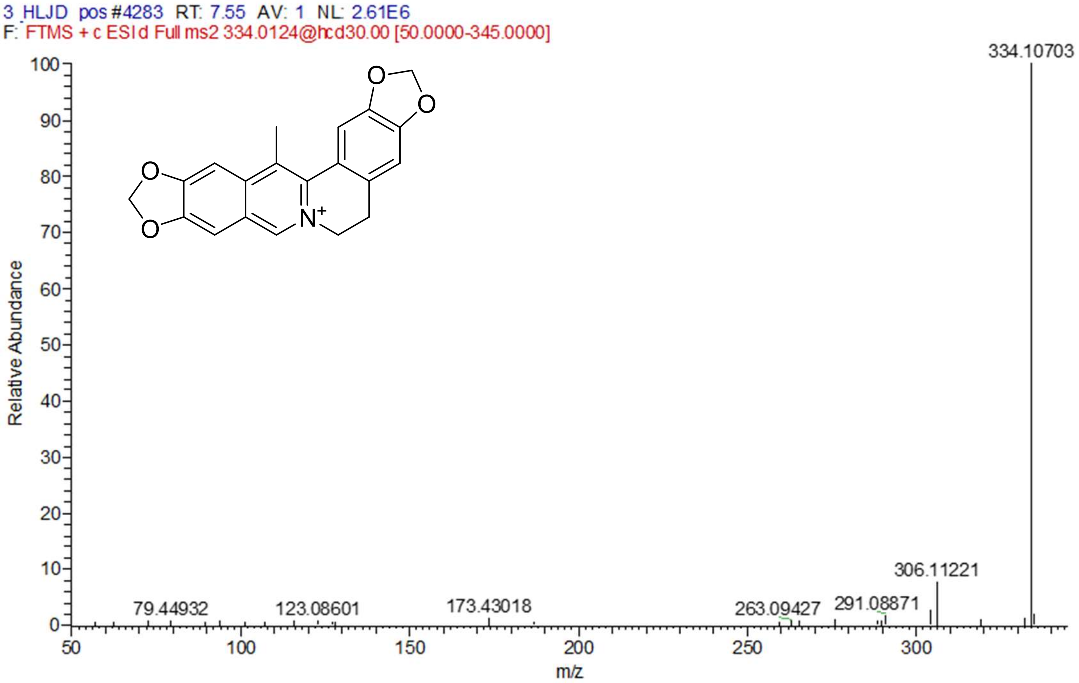

(H) Positive mode spectra for worenine

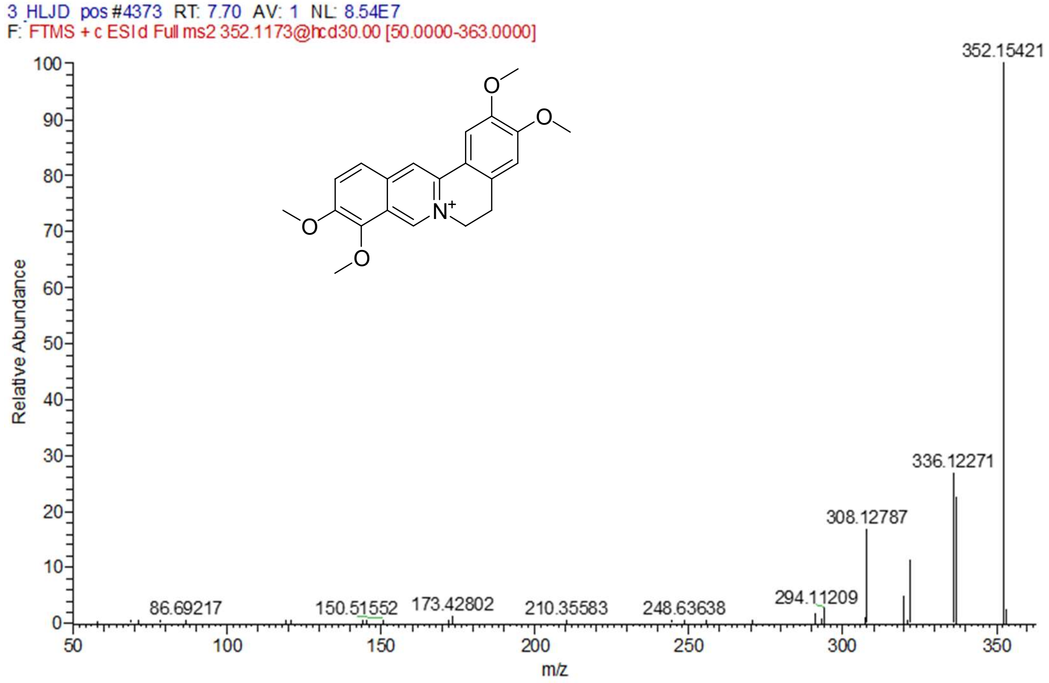

(I) Positive mode spectra for palmatine

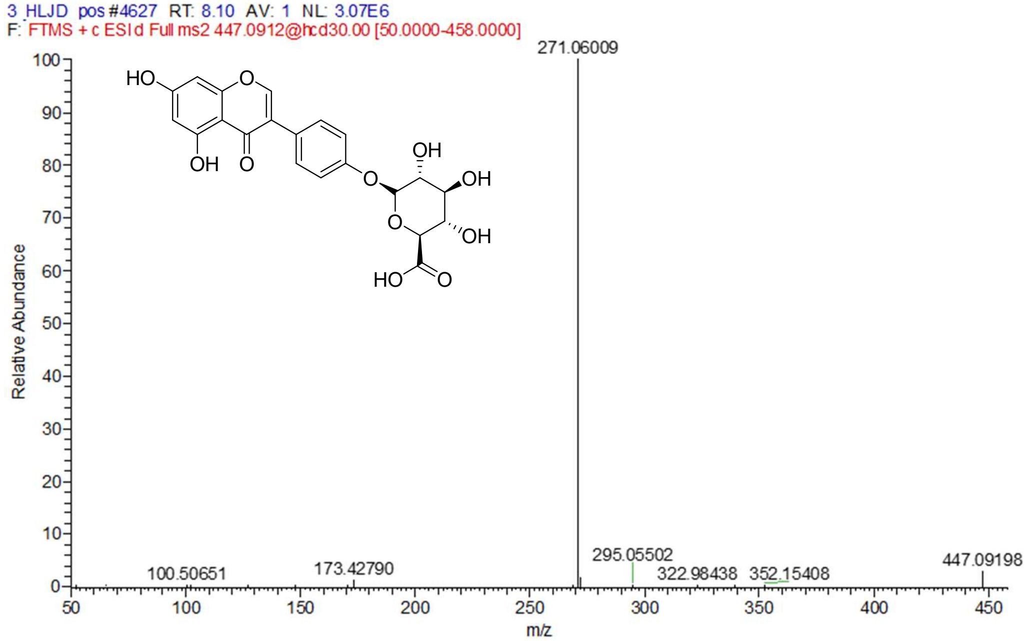

(J) Positive mode spectra for genistein 4'-O-glucuronide

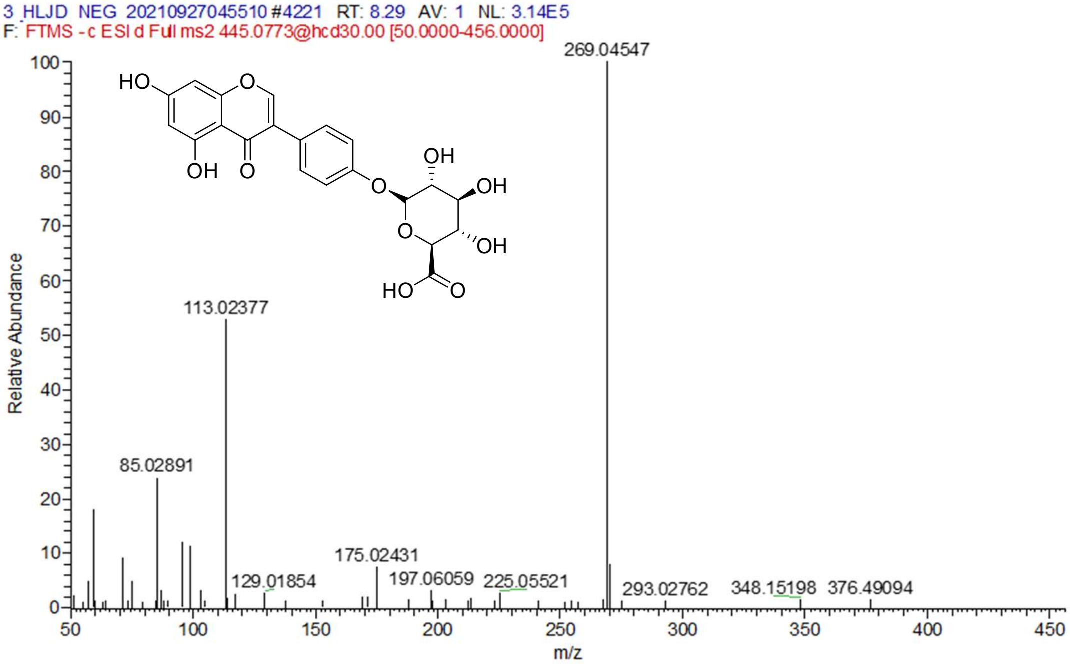

(K) Negative mode spectra for genistein 4'-O-glucuronide

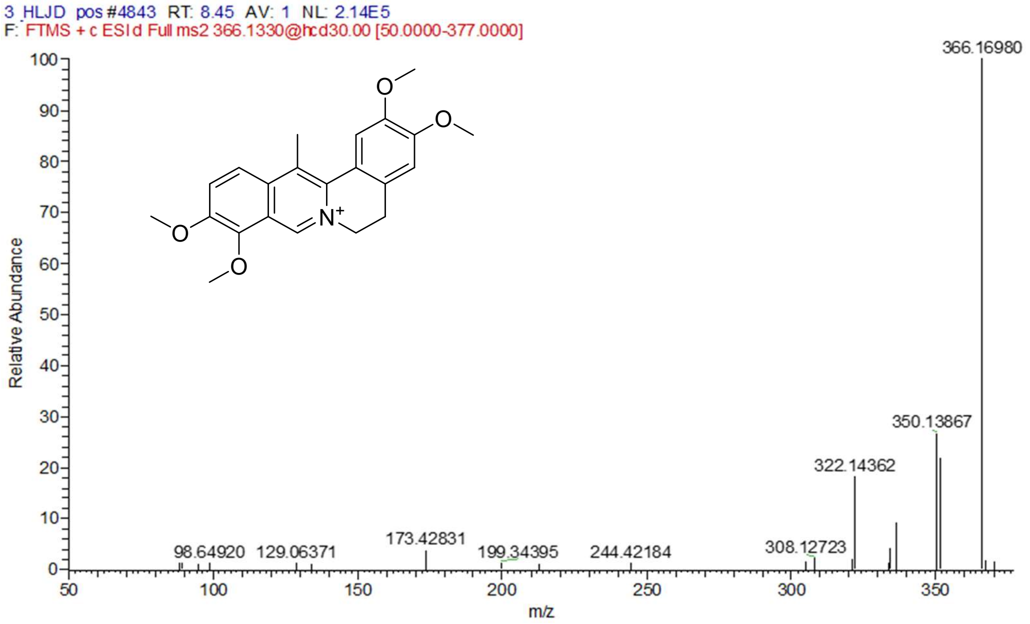

(L) Positive mode spectra for dehydrocorydaline

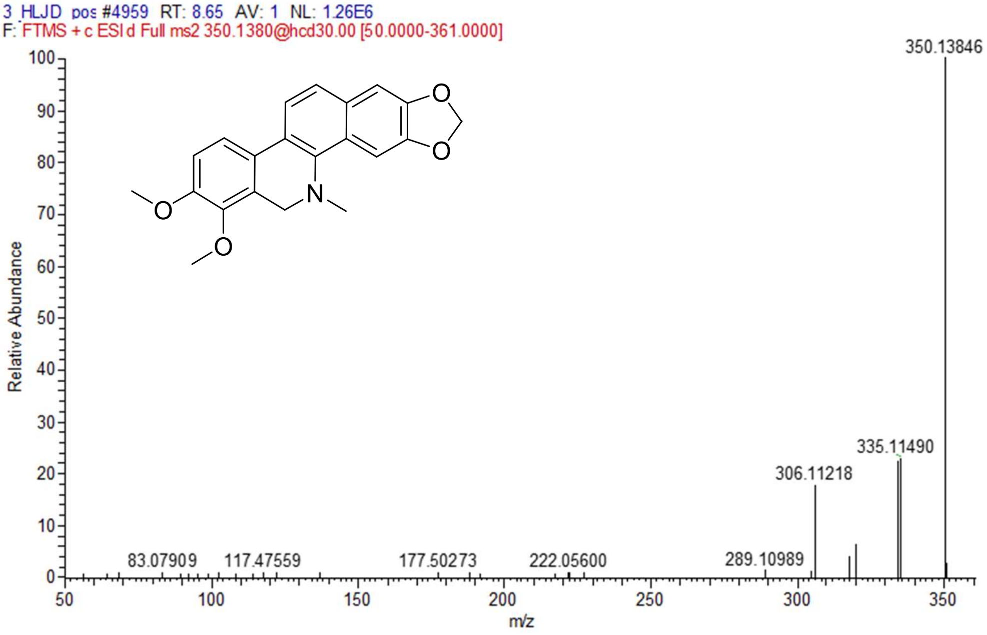

(M) Positive mode spectra for dihydrochelerythrine

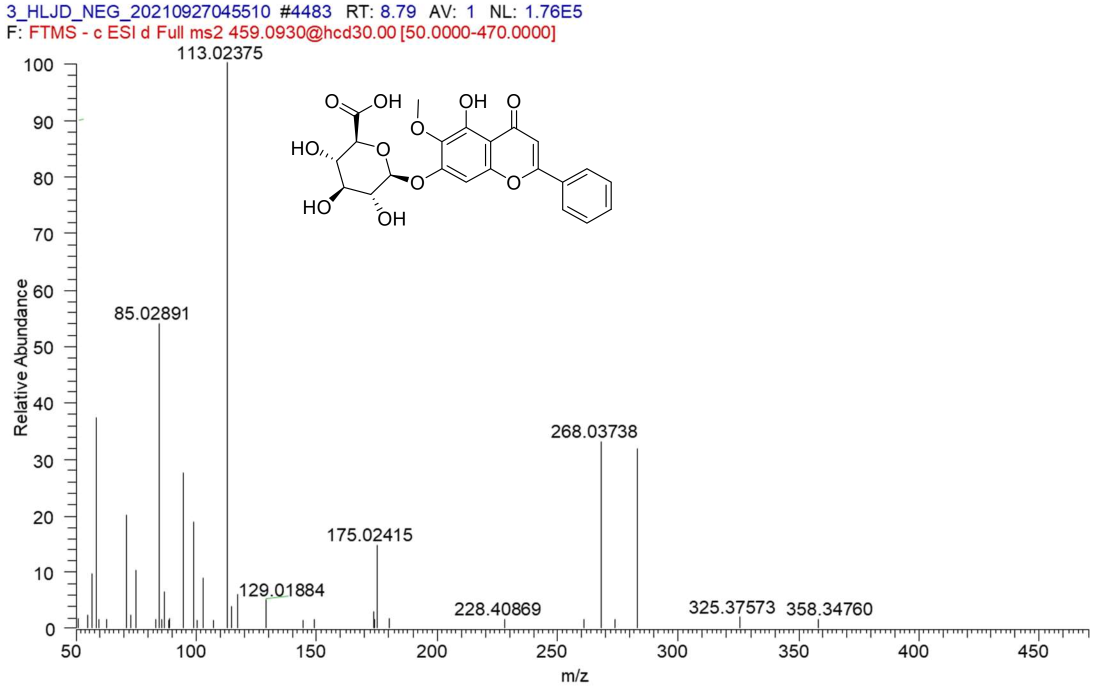

(N) Negative mode spectra for oroxylin A-7-O-β-D-glucuronide

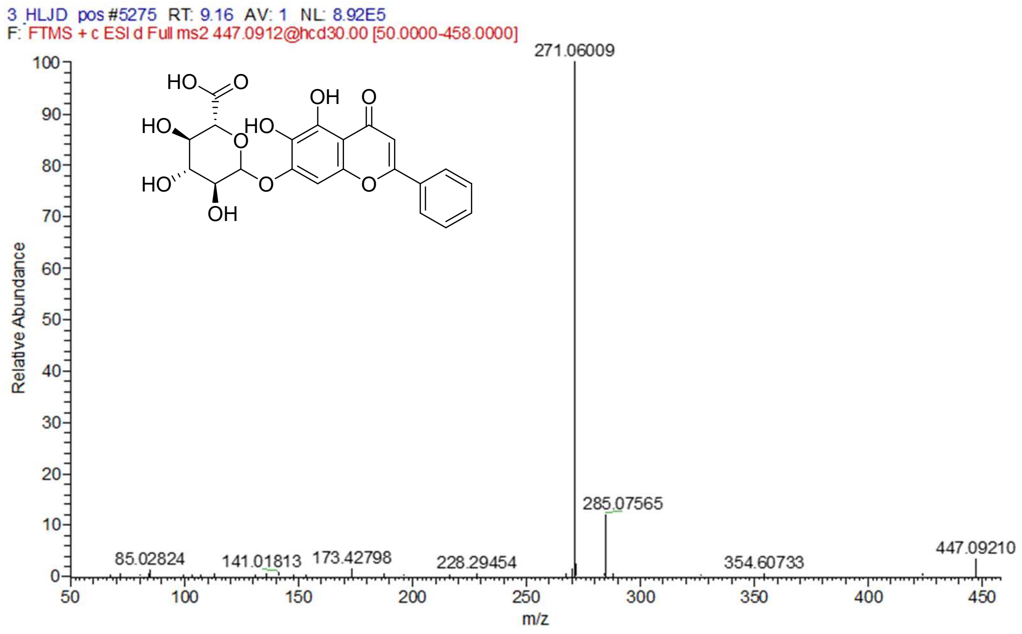

(O) Positive mode spectra for apigenin 7-O-glucuronide

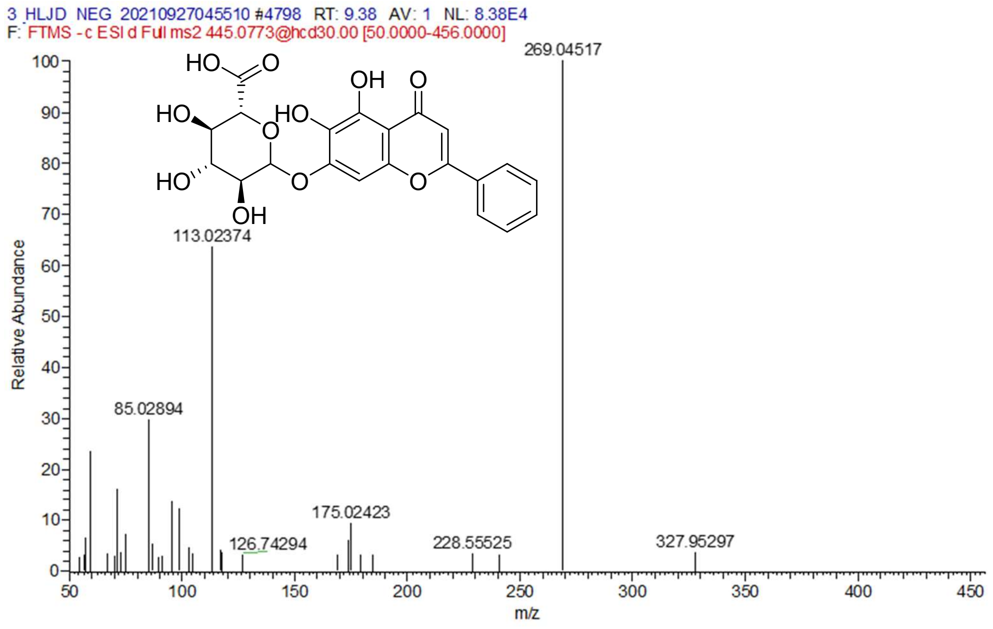

(P) Negative mode spectra for apigenin 7-O-glucuronide

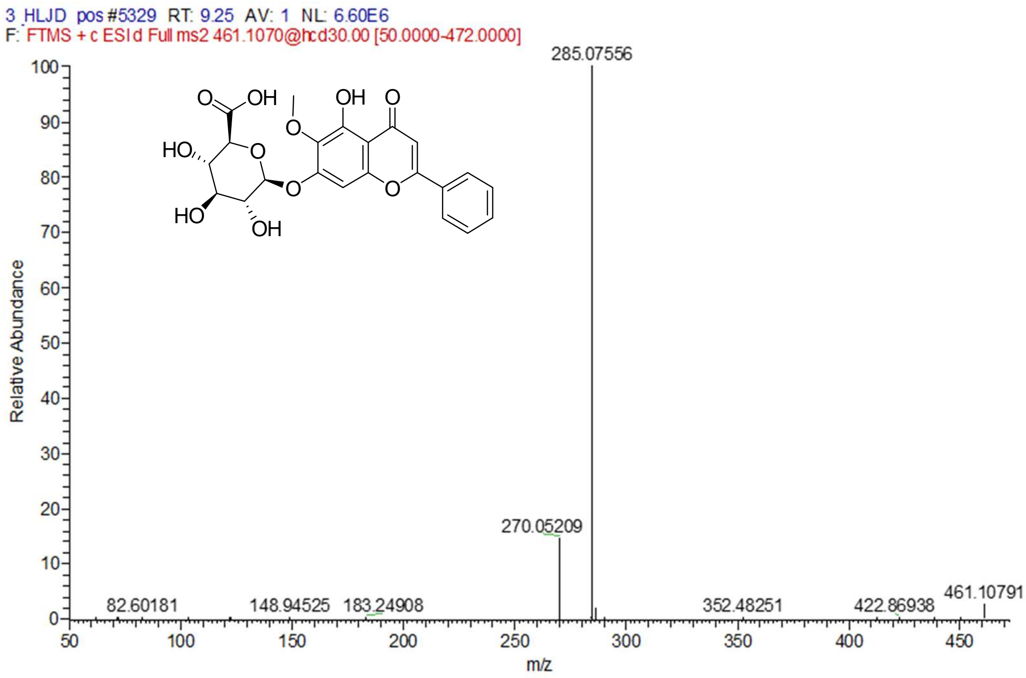

(Q) Positive mode spectra for wogonoside

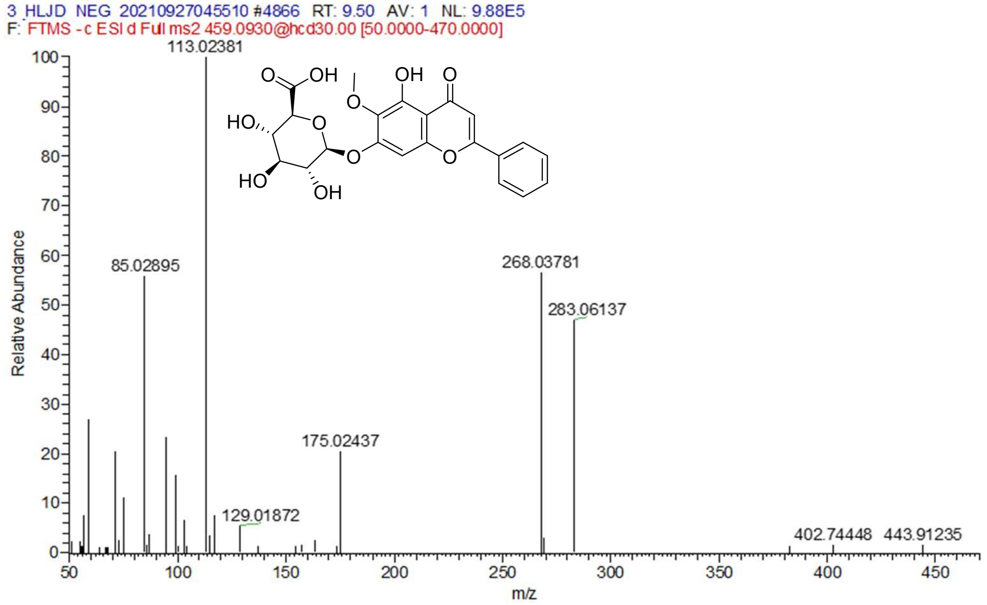

(R) Negative mode spectra for wogonoside

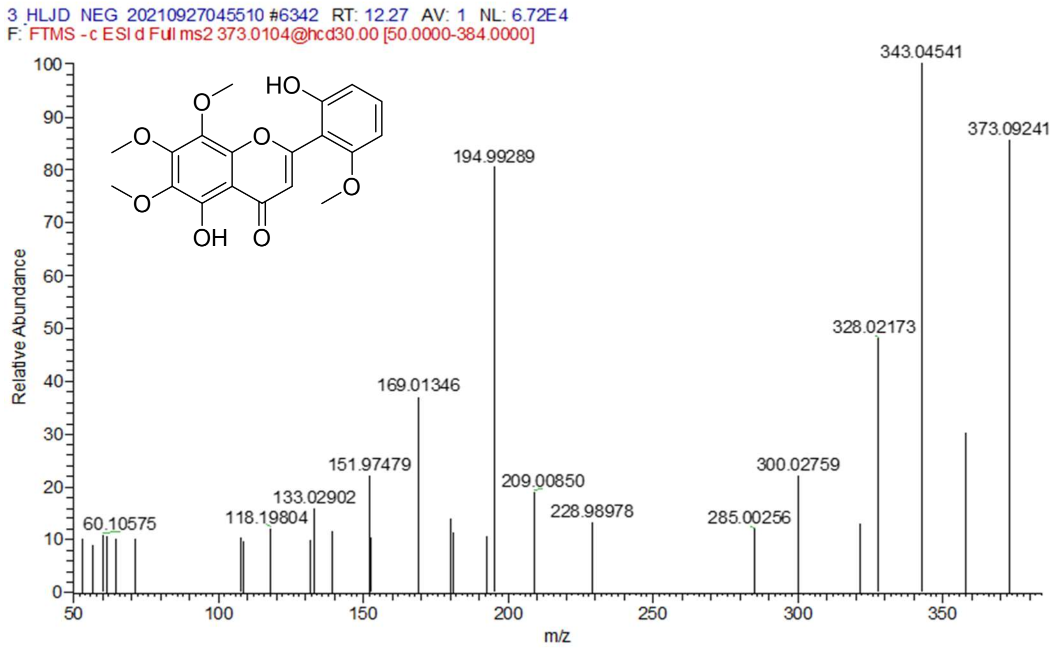

(S) Negative mode spectra for skullcapflavon II

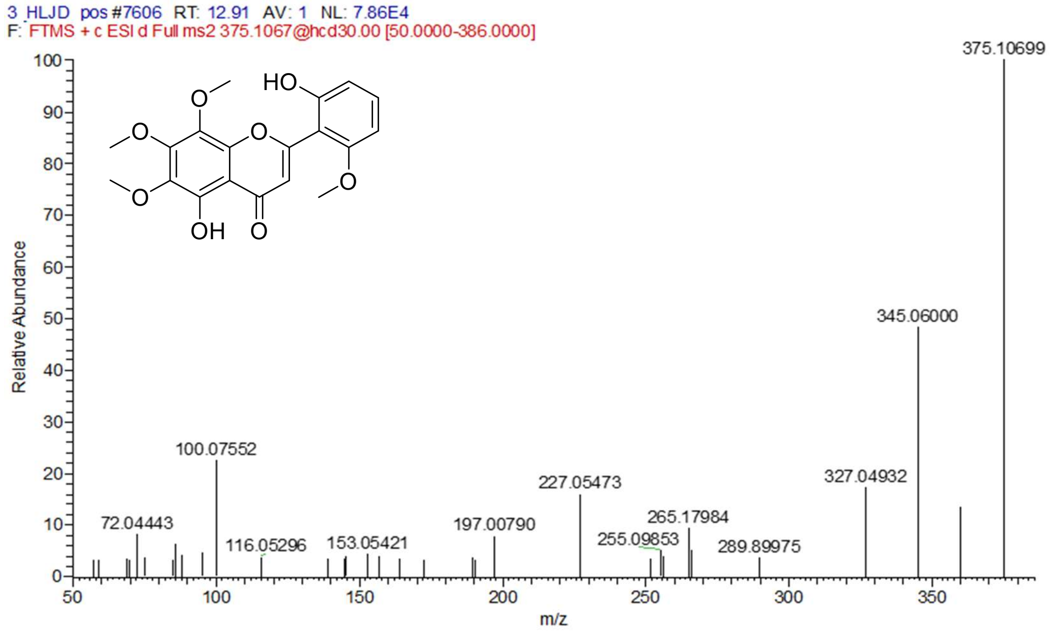

(T) Positive mode spectra for skullcapflavon II

Supplementary Figure 9. Extracted ion chromatogram (EIC/XIC) of the 15 Huanglian Jiedu decoction (HLJDD) components dissociated from the CMs@HT22-MBs

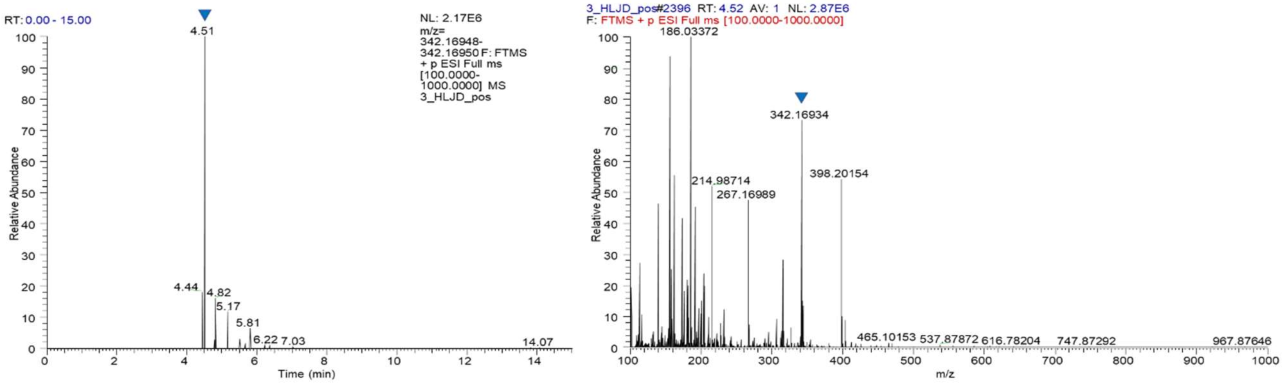

(A) Positive mode spectra for phellodendrine

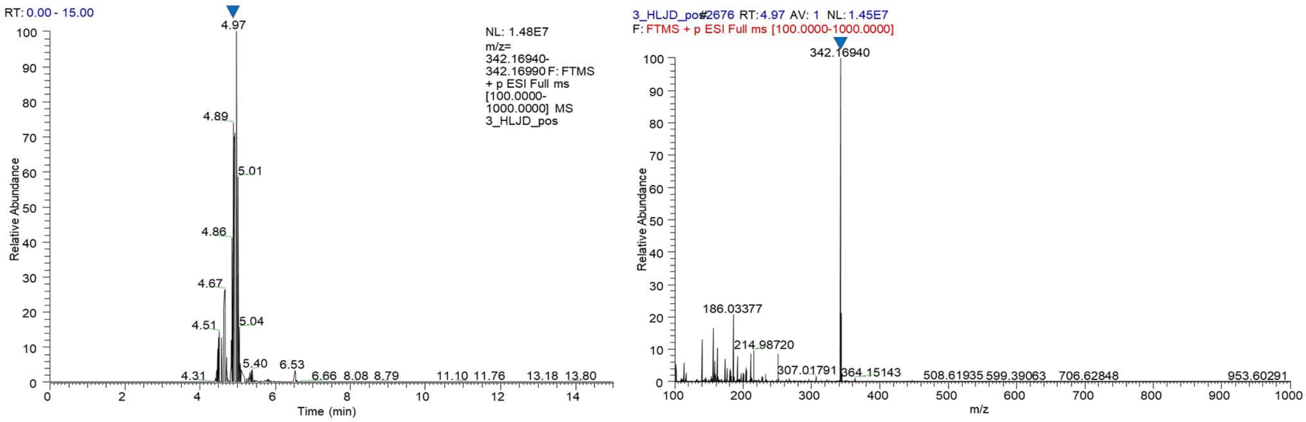

(B) Positive mode spectra for magnoflorine

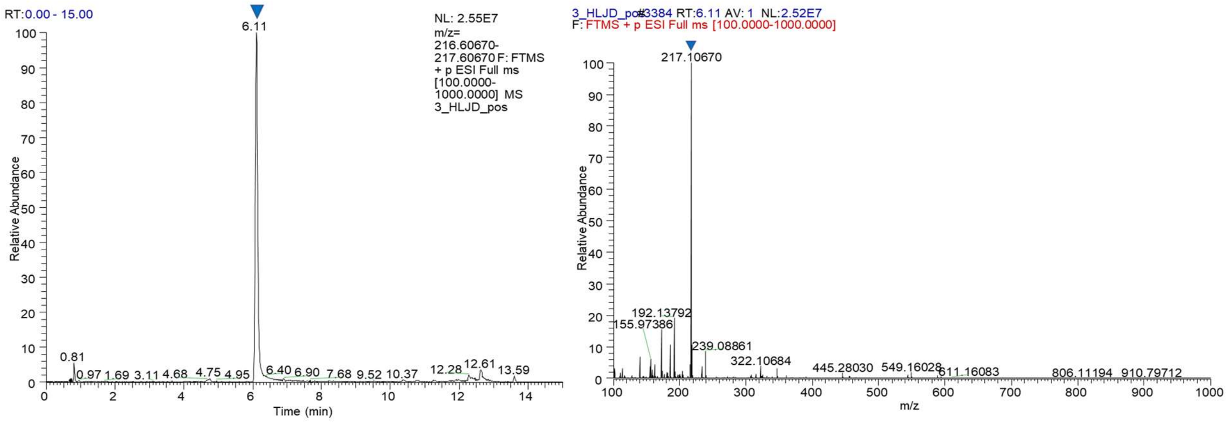

(C) Positive mode spectra for 2,3,4,9-tetrahydro-1H-β-carboline-3-carboxylic acid

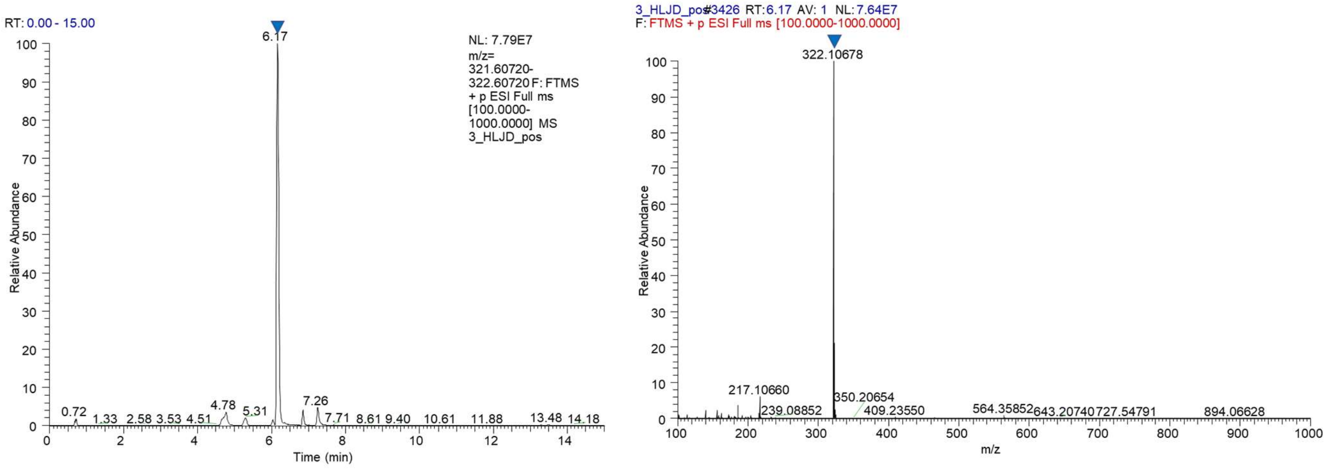

(D) Positive mode spectra for groenlandicine

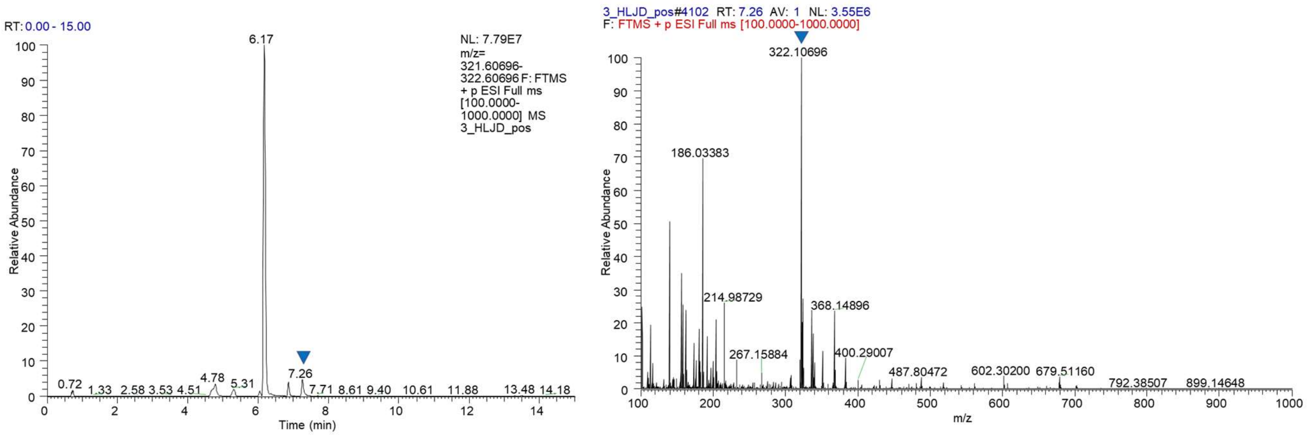

(E) Positive mode spectra for berberrubine

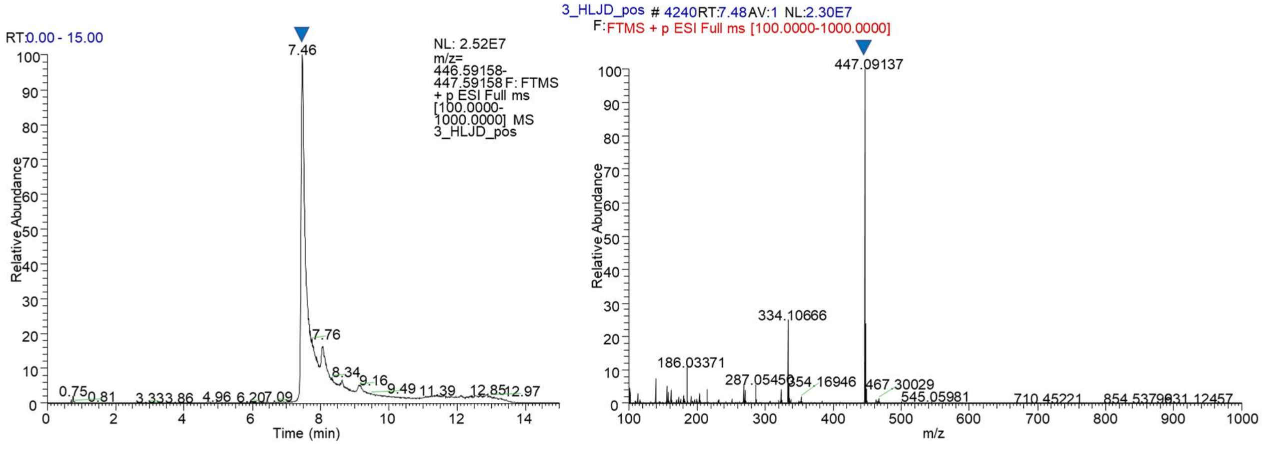

(F) Positive mode spectra for baicalin

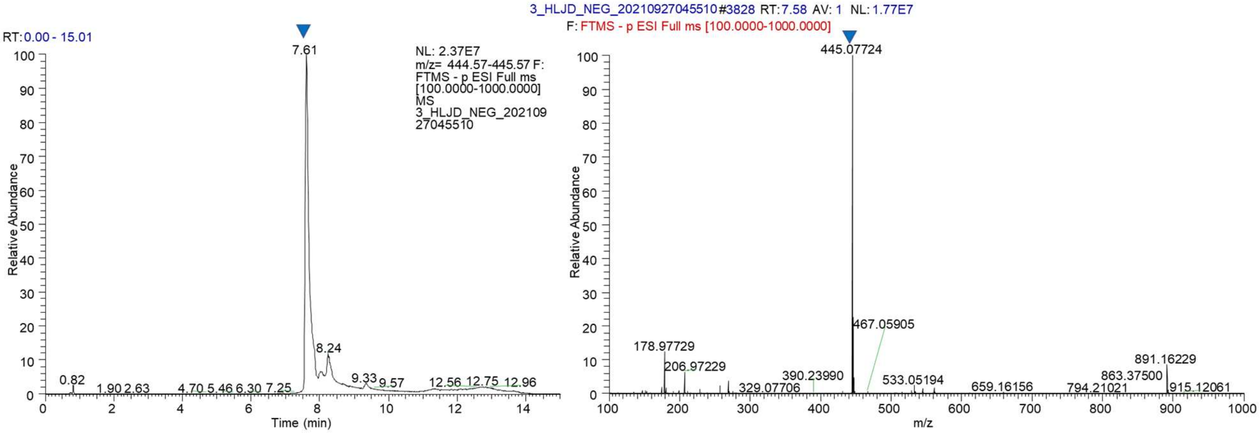

(G) Negative mode spectra for baicalin

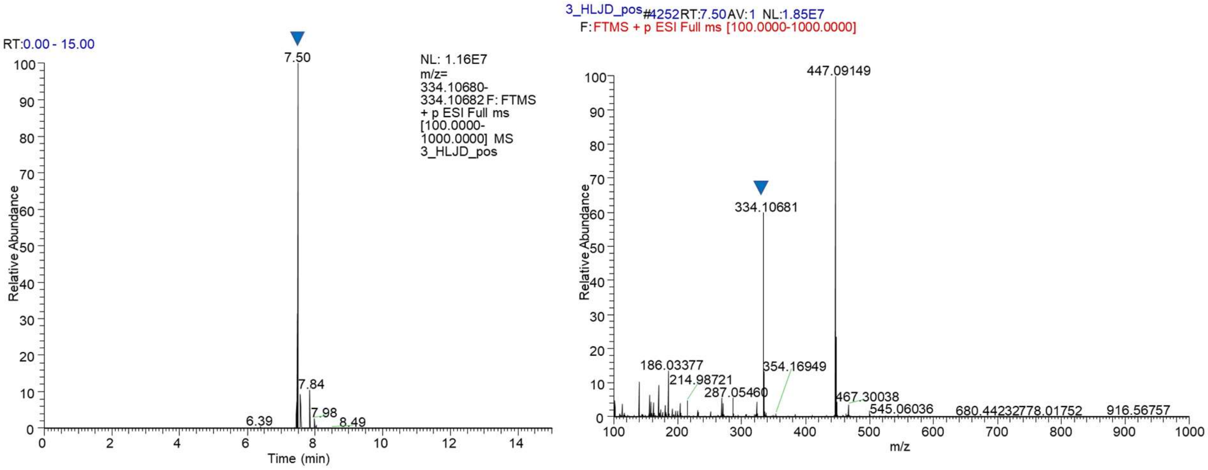

(H) Positive mode spectra for worenine

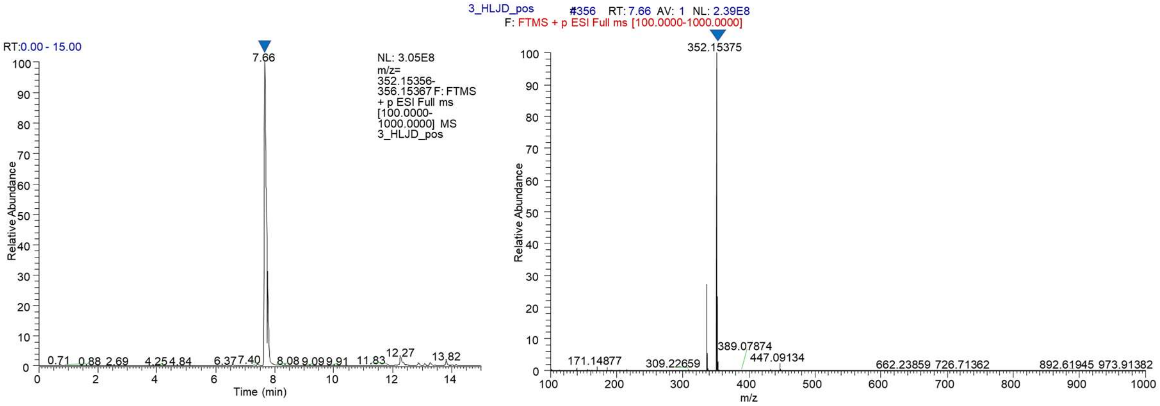

(I) Positive mode spectra for palmatine

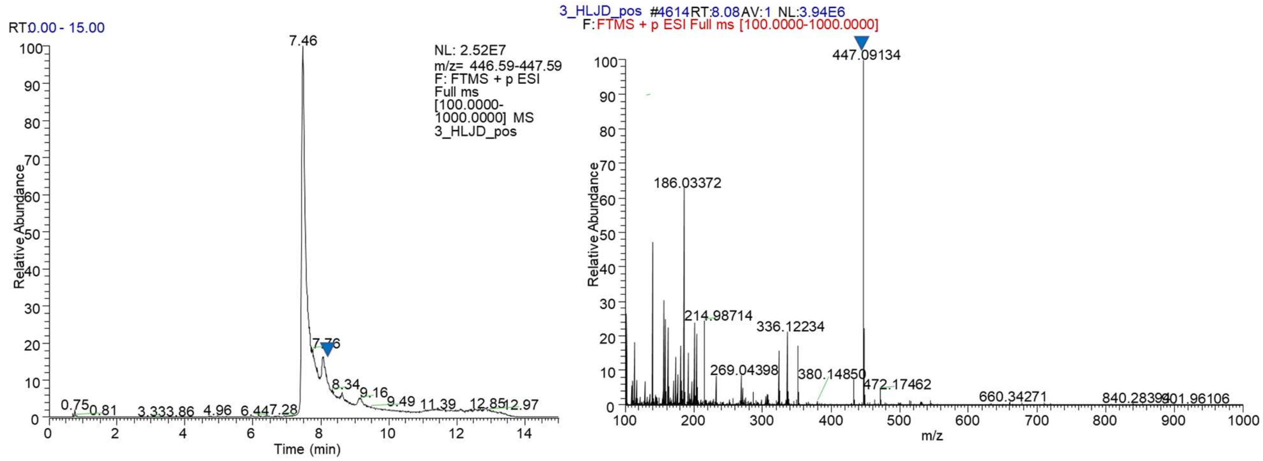

(J) Negative mode spectra for genistein 4'-O-glucuronide

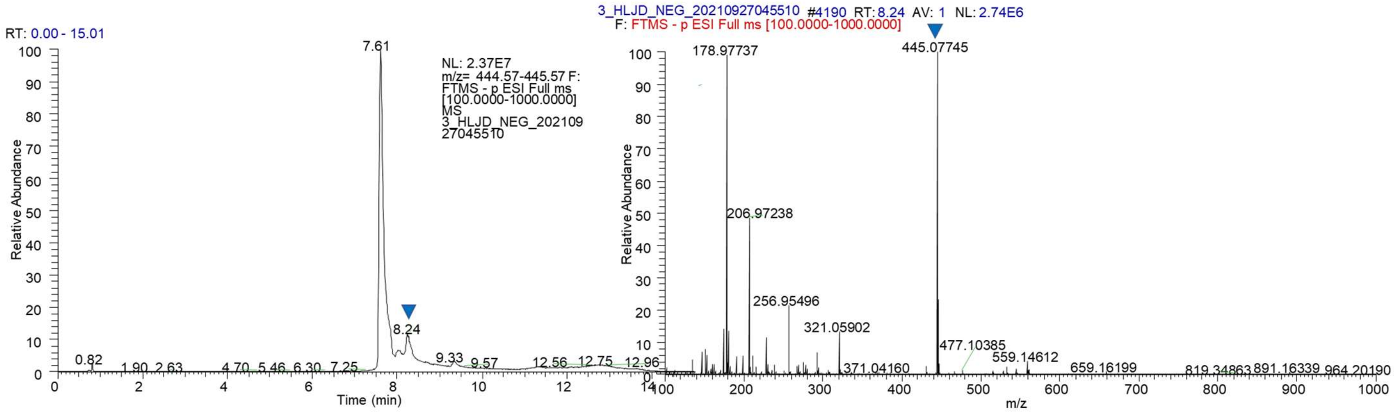

(K) Positive mode spectra for genistein 4'-O-glucuronide

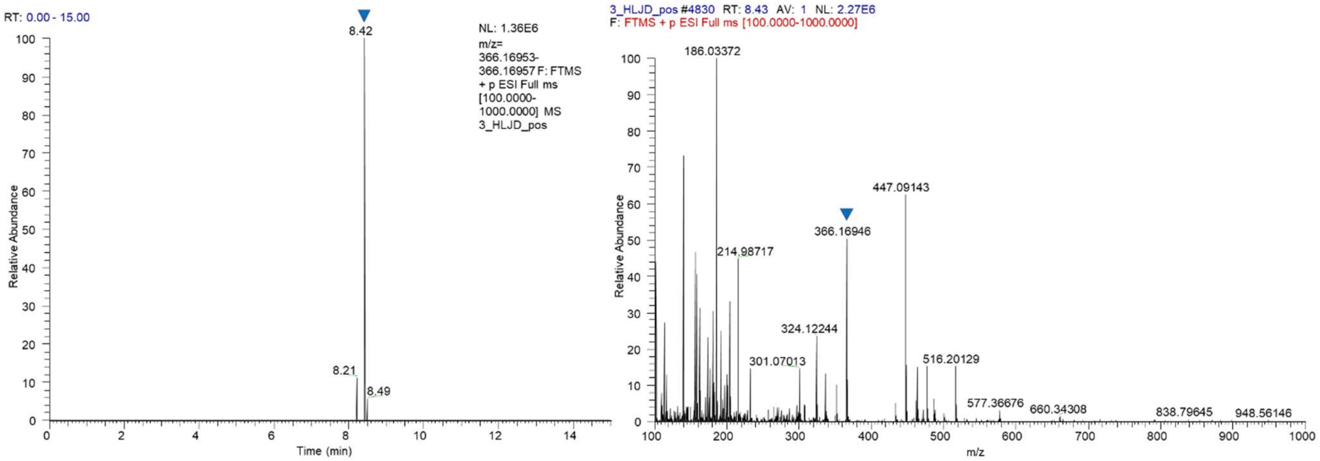

(L) Positive mode spectra for dehydrocorydaline

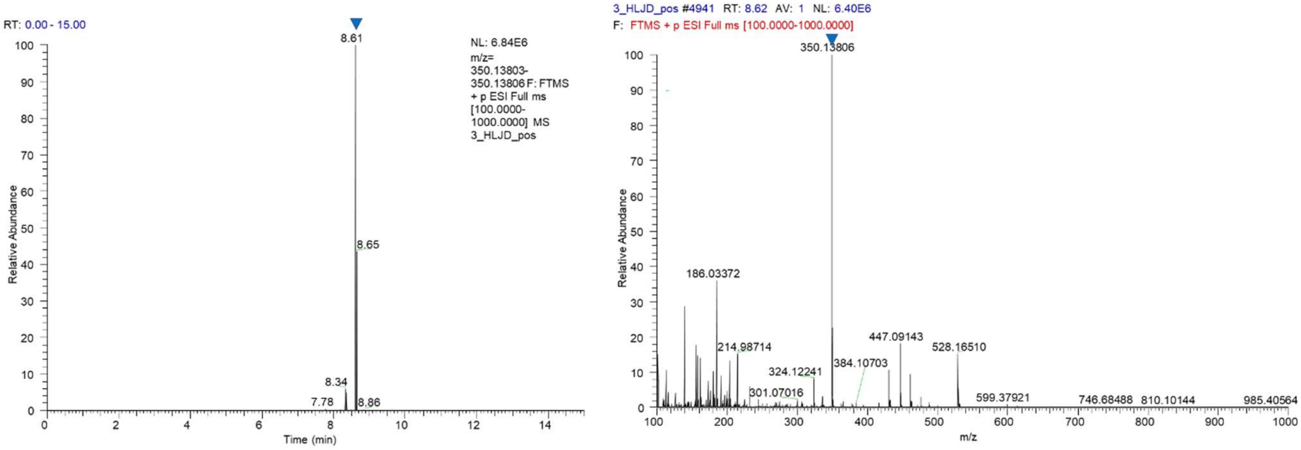

(M) Positive mode spectra for dihydrochelerythrine

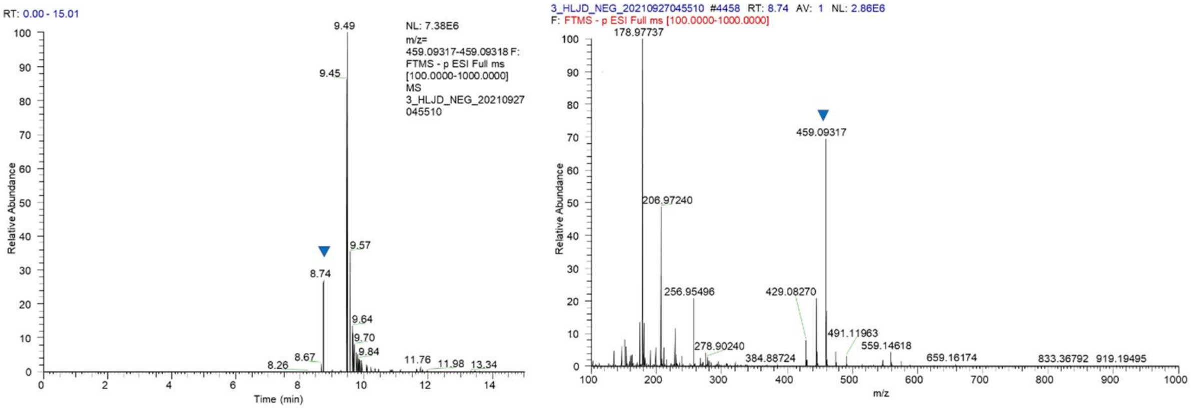

(N) Negative mode spectra for oroxylin A-7-O-β-D-glucuronide

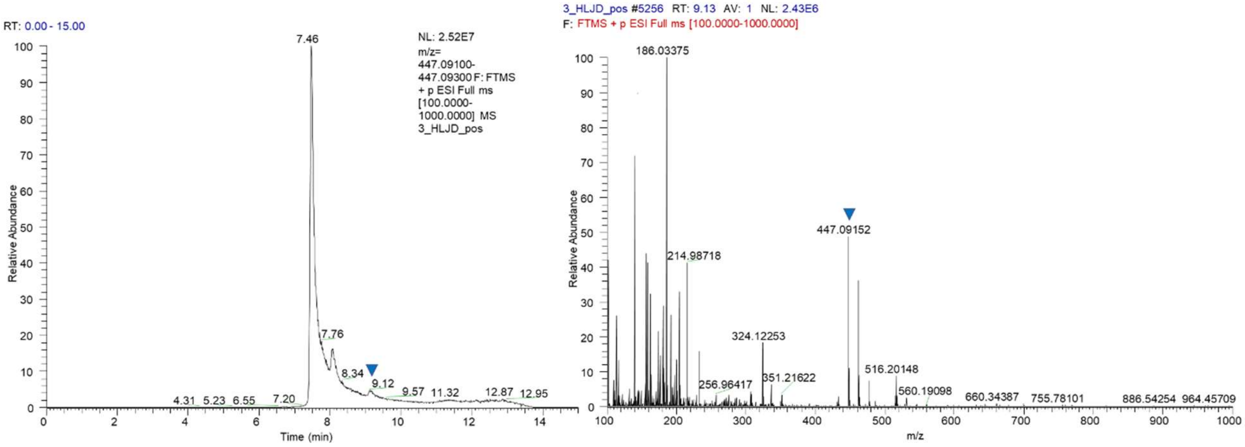

(O) Positive mode spectra for apigenin 7-O-glucuronide

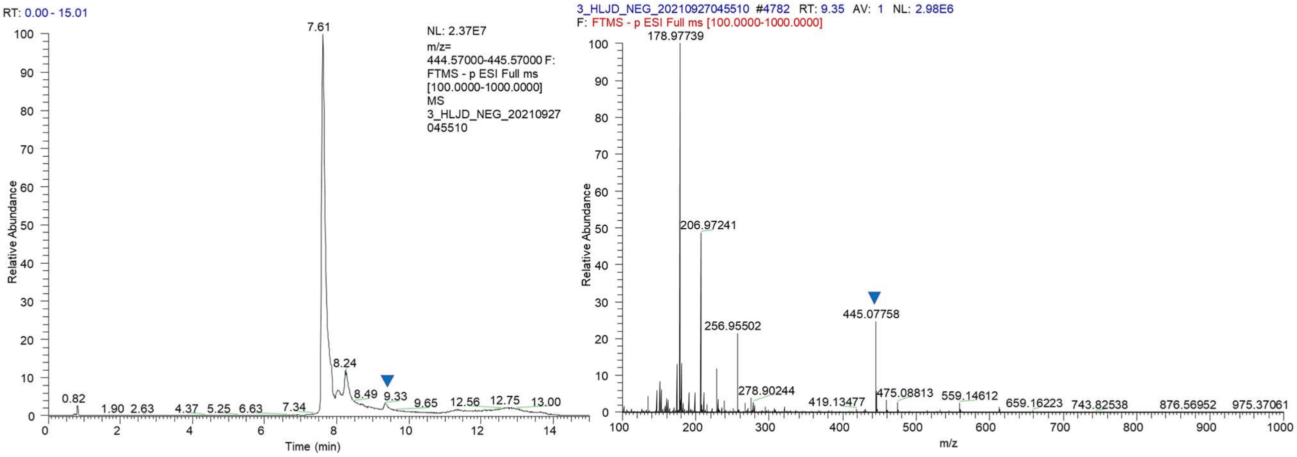

(P) Negative mode spectra for apigenin 7-O-glucuronide

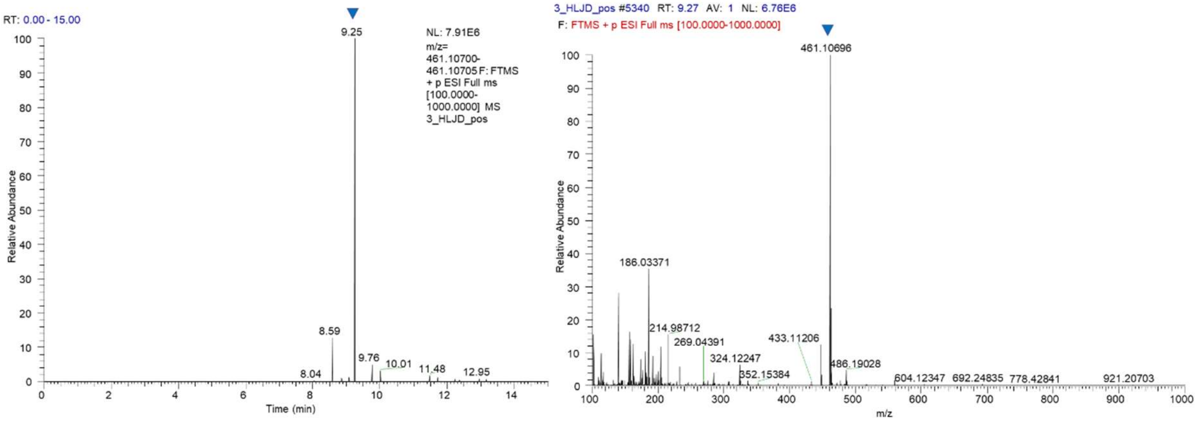

(Q) Positive mode spectra for wogonoside

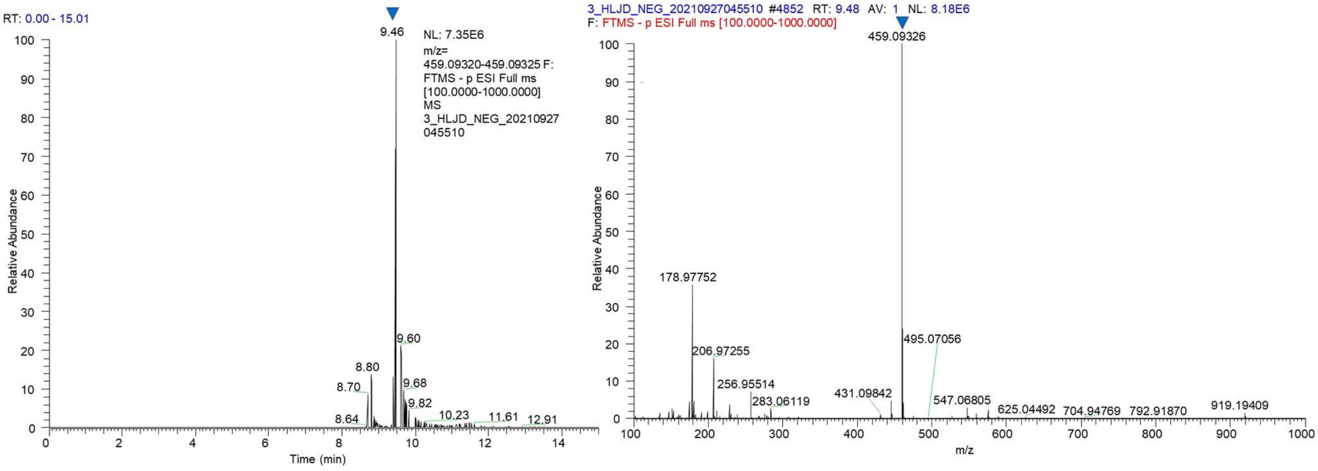

(R) Negative mode spectra for wogonoside

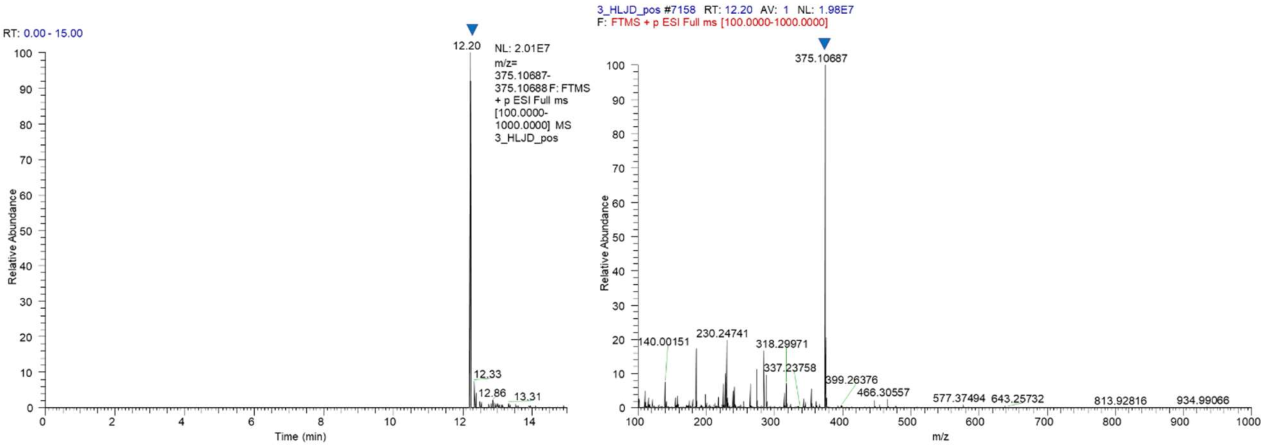

(S) Positive mode spectra for skullcapflavon II

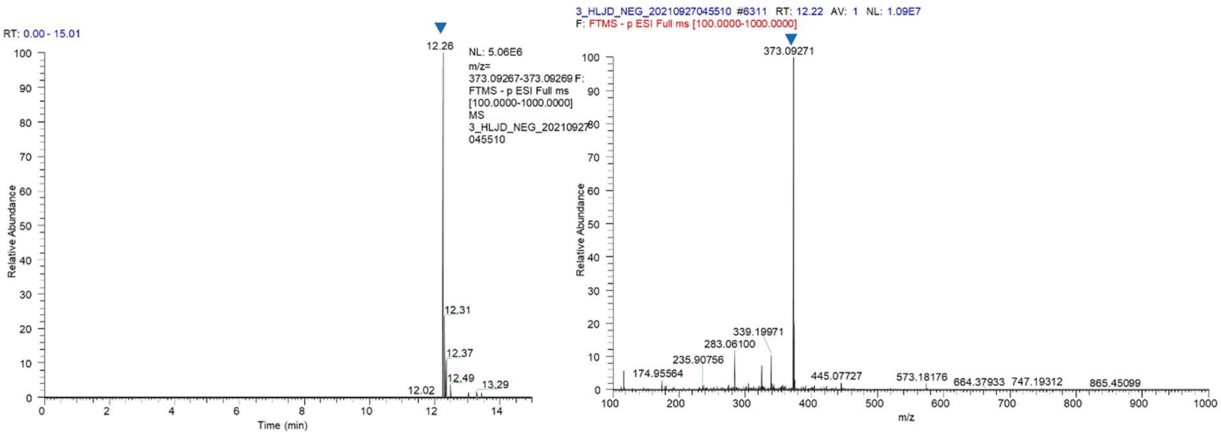

(T) Negative mode spectra for skullcapflavon II

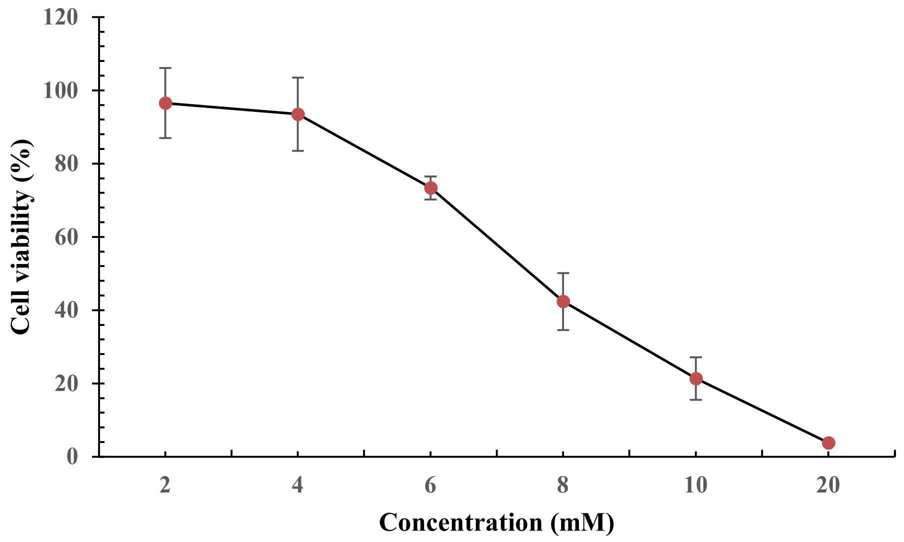

Supplementary Figure 10. Effects of different concentrations of glutamate on the viability of HT22 cells. These cells were treated with various concentrations of glutamate using a CCK-8 viability assay. The HT-22 cells exhibited a significant reduction in cell viability with increasing glutamate concentration. When the glutamate concentration was 8 mM, cell viability was nearly 42% ($p < 0.01$), which is lower than that of the untreated cells. When the glutamate concentration was 10 mM, the viability of the HT-22 cells decreased by nearly 80% ($p < 0.01$) compared to that of untreated cells. When the concentration of glutamate was increased to 20 mM, the cell survival rate was only 4%. The median lethal dose (LD50) of glutamate for HT-22 cells in this experiment was 7.0 mM
